# miR-12135 ameliorates liver fibrosis accompanied with the downregulation of integrin subunit alpha 11

**DOI:** 10.1016/j.isci.2023.108730

**Published:** 2023-12-14

**Authors:** Motofumi Kumazoe, Emi Miyamoto, Chihiro Oka, Miyuki Kondo, Ren Yoshitomi, Hiroaki Onda, Yu Shimada, Yoshinori Fujimura, Hirofumi Tachibana

**Affiliations:** 1 Division of Applied Biological Chemistry, Department of Bioscience and Biotechnology, Faculty of Agriculture, Kyushu University, Fukuoka 819-0395, Japan

**Keywords:** Hepatology, Physiology, Molecular biology

## Abstract

Cirrhosis is becoming one of the most common diseases worldwide. Abnormal upregulation of transforming growth factor β (TGF-β) signaling plays a pivotal role in the excess activation of hepatic stellate cells. However, an efficient countermeasure against abnormal hepatic stellate cell activation is yet to be established because TGF-β signaling is involved in several biological processes. Herein, we demonstrated the antifibrotic effect of miR-12135, a microRNA with unknown function upregulated by isoflavone. Comprehensive transcriptome assay demonstrated that miR-12135 suppressed Integrin Subunit Alpha 11 (ITGA11) and that ITGA11 expression is correlated with alpha smooth muscle actin expression in patients with cirrhosis. miR-12135 suppressed the expression level of ITGA11 and liver fibrosis. Importantly, ITGA11 is overexpressed in activated hepatic stellate cells, whereas ITGA11 knockout mice are viable and fertile. In conclusions, the miR-12135/ITGA11 axis can be an ideal therapeutic target to suppress fibrosis by precisely targeting abnormally upregulated TGF-β signaling in hepatic stellate cells.

## Highlights

• miR-12135 suppressed ITGA11 in abnormally activated hepatic stellate cells
• miR-12135 ameliorated TGFβ-induced fibrosis-related genes in hepatic stellate cells
• miR-12135 improved CDAHFD-induced liver fibrosis
• miR-12135 suppressed phosphorylated-JNK/P-SMAD2/3 signaling

## Graphical abstract

**Figure:**
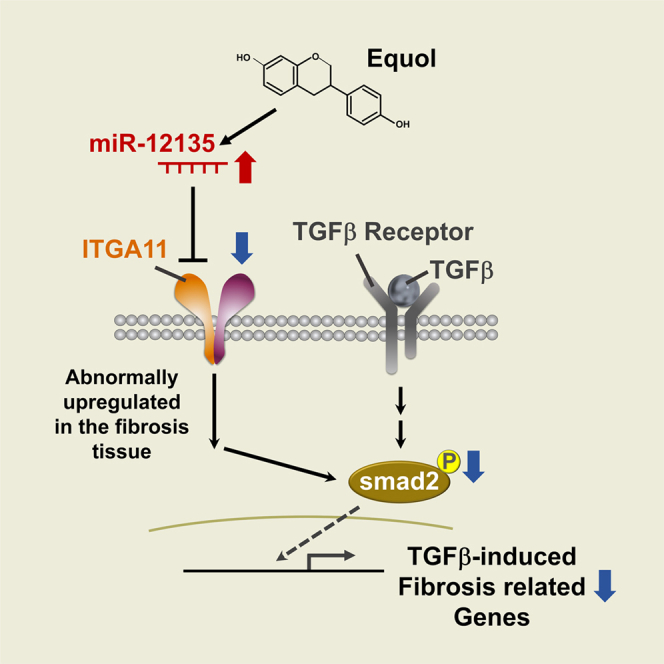

## Introduction

Cirrhosis is becoming one of the most common diseases worldwide.^1^ Although the incidence of virus-induced cirrhosis has decreased due to advances in antiviral therapy,^2^ the incidence of non-alcoholic steatohepatitis (NASH) is rapidly increasing due to lifestyle-related factors, such as the Western diet and obesity.^3^ Several complicated processes are involved in the progression of NASH, including chronic inflammation induced by hyperlipidemia,^3^ triacylglycerol (TG) accumulation,^4^ endoplasmic reticulum stress (ER),^5^ and fibrosis.^6^ Current therapeutic strategies focus on the improvement of glucose intolerance,^7^ and no measures have been established to treat fibrosis.^8^ Fibrosis in NASH is induced by abnormally upregulated transforming growth factor β (TGF-β), which is produced by several types of cells, including macrophages.^9^ TGF-β stimulates hepatic stellate cell activation, which causes the overproduction of collagen and other types of extracellular matrix components^10^ and can even trigger hepatic cancer.^11^ TGF-β/Smad3 signaling is also involved in systemic insulin resistance by regulating the expression of peroxisome proliferator-activated receptor gamma (PPAR-γ) and PPAR-γ coactivator 1 alpha (PGC-1α). Appropriate regulation of this system is crucial for treating patients with NASH.^10^ Besides the indispensable role of TGF-β in this process, it is also heavily involved in the regulation of several physiological processes, such as immune tolerance and cell differentiation.^12^ Moreover, TGF-β is a multifunctional cytokine that plays a crucial role in wound healing, cell survival, and angiogenesis^10^ by binding to type II TGF-β receptors and recruiting TβT-1 or TGF-β receptor type 1 kinase (ALK5).^10^ Thus, it is not feasible to completely inhibit TGF-β owing to the risk of adverse effects.^13^

MicroRNAs (miRs) are short, non-coding RNAs approximately 20 nucleotides in length.^14^ Recent studies have shown that miRs are involved in various biological processes, including tumor progression,^15^ cell differentiation,^16^ immune regulation,^17^ and fibrosis.^18^ In these processes, miRs directly bind to the 3′ non-translational region (UTR) of their target RNAs and negatively regulate translation by degrading the target mRNAs or suppressing their translation.^19^ A recent study demonstrated that approximately 30% of biological processes are regulated by miRs, suggesting their indispensable roles in the body.^20^ Despite the important role of miRs in the regulation of fibrosis, there is no established approach to treat NASH. To the best of our knowledge, there are no reports on the efficacy of miR-12135 in the body.

Equol is a metabolite derived from daidzein (a characteristic polyphenol in soybeans) and is produced in certain microbiomes.^21^ Soymilk has been previously demonstrated to have a prominent liver protective effect in a human trial.^22^ Consistent with these findings, some patients with NASH who have microbiomes that can produce equol have shown good prognosis.^23^ However, little is known about the underlying mechanism, and no reports have discussed the inhibitory effect of equol on TGF-β-induced fibrotic signaling or elucidated the detailed mechanisms. Moreover, the role of equol-induced miRs in suppressing the fibrotic process in NASH remains unknown. Additionally, there are no reports explaining why these beneficial effects have been reported only in humans. Therefore, no relationship between miR-12135, a characteristic human miR, and equol has been revealed.

In this study, we showed that equol suppressed fibrosis-related gene expression and induced miR-12135 with unknown function. We also confirmed that miR-12135 suppressed integrin subunit alpha 11 (ITGA11) in abnormally activated hepatic stellate cells, and miR-12135 also ameliorated TGF-β-induced fibrosis-related genes, including alpha smooth actin (ASMA) and collagen I expression, in hepatic stellate cells. Interestingly, ITGA11 was upregulated by TGF-β. Consistent with an *in vitro* experiment, ITGA11 was overexpressed in the activated hepatic stellate cells of mice under a choline-deficient, L-amino acid-defined, high-fat diet (CDAHFD). Moreover, in hepatic tissues derived from patients with NASH, ITGA11 expression levels correlated with ASMA expression. We also revealed that ITGA11 suppression also inhibited TGF-β-induced fibrosis-related gene expression. Moreover, miR-12135 improved CDAHFD-induced liver fibrosis accompanied with the suppression of CDAHFD-induced upregulation of phosphorylated SMAD2/3 and JNK levels in the activated hepatic stellate cells. Importantly, ITGA11 was overexpressed in abnormally activated hepatic stellate cells, whereas ITGA11 knockout mice are viable and fertile.^24^ In summary, miR-12135 that was upregulated by equol suppressed liver fibrosis and downregulated ITGA11 which was increased in activated hepatic stellate cells. Furthermore, miR-12135 suppressed phosphorylated-JNK/P-SMAD2/3 signaling, which plays an important role in the progression of fibrosis. Thus, miR-12135/ITGA11 could be a novel target in the suppression of liver fibrosis.

### Materials and methods

#### Study approval

All human samples were purchased from TissueArray.Com (Derwood, MD, USA), and written informed consent was obtained from each patient accordance with the principles of the Declaration of Helsinki. The detailed etiology and demographics are provided in Table S1. All animal experiments were carried out in accordance with the notifications (no. 6) and regulations in Japan (no. 105). All studies were approved by the Kyushu University Animal Care and Use Committee (Fukuoka, Japan). All experiment procedures were performed in accordance with guidelines.

#### Materials and reagents

The detail Cat number is shown as **Resource Table**. Equol was purchased from TIC (Tokyo Chemical Industry Co. Ltd., Tokyo, Japan) and dissolved in DMSO (50 mM). Fetal bovine serum (FBS) was from Sigma Aldrich (St Louis, MO, USA). Streptomycin and penicillin G were from Meiji Pharmaceutical Co. (Tokyo, Japan). Dulbecco’s modified Eagle’s medium (DMEM) was from Fujifilm Wako (Osaka, Japan). The miR-12135 and adenylated miR-12135 mimics were from Sigma Aldrich (Detail information of sequence is provided as Table S2). SiRNA-ITGA11 (siRNAID:154044, pool) was from Dharmacon (Lafayette CO, USA). TGF-β was from R and D systems (McKinley Place, NE, USA). Anti-ITGA11 antibody was from Abcam (ab198826; Cambridge, UK). Anti-ASMA antibody was from Cell Signaling Technology (#48938S Danvers, MA, USA). Anti-col1a1 antibody was obtained from Santa Cruz Biotechnology (sc-59772, Santa Cruz, CA, USA). Anti-phospho-smad2 Ser465/467 antibody was from Cell Signaling Technology (#8828S), while anti-phospho-JNK (T183/Y185) antibody was from R&D Systems, Inc. (AF1205-SP). Anti-Glyceraldehyde-3-phosphate dehydrogenase (GAPDH) antibody were from Cell Signaling Technology (#5174S). Anti-b actin was from Sigma Andrich (A2228). Alexa Fluor 555 labeled goat anti-rabbit antibody Fab Fragment, Alexa Fluor 488 labeled goat anti-mouse antibody Fab Fragment, and Hoechst33342 were purchased from Thermo Fisher Scientific (Waltham, MA, USA). ONE-Glo EX Luciferase Assay Substrate REF:E633A and FuGENE6 Transfection Reagent REF:E269A were purchased from Promega.

#### Cell culture and assay

Human cell line derived from cervical cancer, HeLa cells (ATCC, Manassas, VA, USA), were cultured in DMEM supplemented with penicillin-streptomycin and 10% FBS under 100% humidity and 5% CO_2_ at 37°C. Hepatic stellate cells (LX2 cells) (Merck, German, Darmstadt) were maintained in DMEM supplemented with penicillin-streptomycin and 2% FBS under 100% humidity and 5% CO_2_ at 37°C.

In the NGS analysis for adenylated micro RNAs, HeLa cells were seeded in a 10-mL dish and precultured in 10% FBS DMEM for 24 h, which was then replaced by 2%FBS DMEM with or without 10 μM equol supplementation. The cells were cultured for 24 h and harvested using TRI Reagent (Cosmo Bio, Tokyo, Japan). Hiseq was performed using the Cell innovator (Fukuoka, Japan).

In the real-time PCR analysis of HeLa cells, HeLa cells were seeded in a 12-well plate and precultured for 24 h in 10% FBS DMEM. After preculture, cells were treated with 10 nM miR-12135 mimic and adenylated miR-12135 mimic using the RNAi max (Thermo Fisher Scientific, Added 200 μL of Opti-MEM 1.7 mL + RNAi max 20.4 μL + miR-12135 mimic [10 μM] 10.2 μL) for 48 h and harvested using the TRI Reagent.

In the real-time PCR analysis of LX2 cells for the effect of equol, LX2 cells were seeded in a 12-well plate and precultured for 24 h in 2% FBS DMEM. Afterward, LX2 cells were treated with equol (10 μM) for 24 h and treated with TGF-β (5 ng/mL) for 48 h and harvested using the TRI Reagent.

In the real-time PCR analysis of LX2 cells for the effect of SiRNA-ITGA11, LX2 cells were seeded in a 12-well plate and precultured for 24 h in 2% FBS DMEM. Afterward, LX2 cells were treated with SiRNA-ITGA11 using the RNAi max (Added 200 μL of Opti-MEM 1.7 mL + RNAi max 20.4 μL + SiRNA-ITGA11 [5 μM] 10.2 μL) for 24 h and treated with TGF-β (5 ng/mL) for 48 h and harvested using the TRI Reagent.

In the WB analysis of LX2 cells for the effect of miR-12135 alone, LX2 cells were seeded in a 12-well plate and precultured for 72 h in 2% FBS DMEM. Afterward, LX2 cells were treated with miR-12135 mimic using the RNAi max for 48 h and harvested using the sample buffer.

In the real-time PCR analysis of LX2 cells for the effect of miR-12135 and TGF-β, LX2 cells were seeded in a 12-well plate and precultured for 24 h in 2% FBS DMEM. Afterward, LX2 cells were treated with miR-12135 mimic using the RNAi max for 24 h and treated with TGF-β (5 ng/mL) for 48 h and harvested using the TRI Reagent.

In the fluorescence microscopy analysis for the effect of equol, LX2 cells were seeded in a glass bottom dish (Mastunami, Osaka, Japan) and precultured for 24 h in 2% FBS DMEM. Afterward, LX2 cells were treated with equol (10 μM) for 24 h and treated with TGF-β (5 ng/mL) for 48 h and fixed by 2% paraformaldehyde (PFA) (Fujifilm, Tokyo, Japan). The cells were also treated with 1% FBS-Phosphate Buffered Saline (PBS)-0.2% Tween and incubated with primary antibody (1:200 in 1% FBS 0.1% sodium azide containing PBS for 0.75 h) and washed with PBS 3 times. Dishes were treated with secondary antibody (1; 300 in 1% FBS 0.1% sodium azide containing PBS supplemented with Hoechst33342 [10000 dilution Thermo Fisher Scientific] for 0.75 h.). After washing thrice with PBS, the cells were observed using the BZ-X700 (Keyence) under 40× magnification.

In the western blotting analysis of LX2 cells for the effect of miR-12135 alone, LX2 cells were seeded in a 12-well plate and precultured for 24 h in 2% FBS DMEM. Afterward, LX2 cells were treated with miR-12135 mimic using the RNAi max for 72 h and harvested using lysis buffer (50 mM Tris-HCl, 150 mM NaCl, 50 mM NaF, 30 mM Na_4_P_2_O_7_; 1 mM EDTA supplemented with 1%Triton X-100 and 1 mM phenylmethanesulfonyl fluoride [PMSF] and reactivated 1 mM pervanadate; 2 mg/mL aprotinin).

In the western blotting analysis of LX2 cells for the effect of miR-12135 and TGF-β, LX2 cells were seeded in a 12-well plate and precultured for 24 h in 2% FBS DMEM. After that, LX2 cells were treated with miR-12135 mimic using the RNAi max for 24 h and treated with TGF-β (5 ng/mL) and harvested using lysis buffer as previously described.

In the viable cell analysis of LX2 cells for the effect of miR-12135, LX2 cells were seeded in a 96-well plate and precultured for 24 h in 2% FBS DMEM. After that, LX2 cells were treated with miR-12135 mimic using the RNAi max for 24 h and 48 h and the viable cell numbers were assessed by ATPlite one step (PerkinElmer) as follows by manufacture’s protocol.

In the miR target luciferases assay, HeLa cells were seeded in a 96-well plate and precultured for 24 h. The medium was removed and replaced with 2%FBS DMEM. Afterward, we added luciferase vector with the 3′UTR of ITGA11 with or without the target predicted sequence of miR-12135 (NM_001004439.1, pEZX-MT06 HmiT130500-MT06, from GeneCopoeia) and control luciferase vector without the 3′UTR of ITGA11 (HmiT130500-MT06, from Gene Copoeia) by using Fugene 6 and transfected with miR-12135 mimic (10 nM) or Negative control (NC) mimic (10 nM) by RNAimax. After 48 h, the supernatants were removed, ONE-Glo EX Luciferase Assay Substrate was added, and the cells were measured using the plate reader Envision.

In the miR-12135 inhibitor assay, LX2 cells were seeded at glass bottom dish (2%FBS DMEM, 1 mL/dish) and precultured for 24 h. After preculture, cells were treated with NC mimic or miR-12135 inhibitor (10 nM, Ambion, mirVana, Cat#4464084) by using RNAimax ([RNAimax 20.4 μL + Optimem 850 μL] + [miR-12135 inhibitor (10 μM) 10.2 μL + Optimem 850 μL] 200 μL/well). Afterward, 6 h later, cells were treated with 10 μM equol, and 24 h later, cells were treated with TGF-β (5 ng/mL) for 48 h. After treatment, cells were fixed using 2% PFA and treated with 1% FBS-PBS-0.2% Tween and incubated with primary antibody (1:200 in 1% FBS 0.1% sodium azide containing PBS for 0.75 h) and washed by PBS 3 times. Dishes were treated with secondary antibody (1:300 in 1% FBS 0.1% sodium azide containing PBS supplemented with Hoechst33342 [10000 dilution Thermo Fisher Scientific] for 0.75 h). After washing thrice with PBS, the cells were observed by using BZ-X700 (Keyence) under 40× magnification.

#### Western blot analysis

Harvested cell lysate (approximately 10 μg protein) was added using a sample buffer (1% sodium dodecyl sulfate [SDS], 10% glycerol, 0.001% bromophenol blue, 100 mM Tris-HCl buffer [pH 6.8] supplemented with 0.05% mercaptoethanol). The solutions were heated using a heat block (for 5 min at 95°C), stored at −30°C, then electrophoresed by using SDS-polyacrylamide gels (SDSPAGE) (0.03A, 1.5 h) and nitrocellulose membranes (100 V, 1 h). Nitrocellulose membranes were blocked using blocking buffer with Tween 20-TBS (TTBS) containing 2.5% BSA for 1 h, then incubated with the primary antibody (1:3000 except for loading control). Afterward, these were diluted using blocking buffer overnight, washed with TTBS, then incubated with horseradish peroxidase-conjugated secondary antibodies (anti-rabbit [1:10,000] or anti-mouse [1:10,000]) for 1 h. After membranes were washed with TTBS, membranes were detected with an enhanced chemiluminescence system (Lumigen, ECL Ultra, TMA-6) using the Fusion System (Vilber-Lourmat).

Band intensity was evaluated by using Kyplot (KyensLab Inc., Tokyo, Japan).

#### Animals and animal experiments

Because the pharmacological effects of miR-12135 were not predicted, group size was not calculated. All mice used in this study were maintained in an approximately humid (60%), temperature-controlled (20°C) room with a 12-h light–dark cycle (dark from 8 p.m. to 8 a.m.). Mice were allowed *ad libitum* accessed to diet and drinking water. We confirm that the study is in accordance with the ARRIVE guidelines.

In the NASH model (immunohistochemistry staining in Figure 3), 5-week-old male C57BL/6J mice (2 groups, 3 mice each) were purchased from Kyudo (Saga, Japan) and acclimated for 1 week by being fed MF diet (KBT Oriental, Saga, Japan).

At 6 weeks of age, mice were randomly entered into 2 groups: Gp1 was fed with MF diet (n = 3), while Gp2 was fed with CDAHFD (Research Diet, A06071302) (n = 3). After 4 weeks, all mice were sacrificed under isoflurane vapor. The organs were harvested and used for analysis. No mouse was excluded in any group.

In the NASH model (miR-12135 treatment), 5-week-old male C57BL/6J mice (4 groups, 5 mice each; total of 20) were purchased from Kyudo (Saga, Japan) and acclimated for 1 week by being fed MF diet (Saga, Japan).

At 6 weeks of age, mice were randomly entered into 4 groups: Gp1 was fed with MF diet (n = 5), Gp2 was fed with CDAHFD and not treated with targeting miRNA (n = 5), Gp3 was fed with CDAHFD and treated with miR-12135 (n = 5), Gp4 was fed with CDAHFD (and treated with adenylated miR-12135 (adenylated) (n = 5). At days 5, 8, 12, 15, and 19, the mice were given each miR (3 nmol/mouse intraperitoneally in Atelogene [Koken, Tokyo, Japan]). At day 20, all mice were sacrificed under isoflurane vapor. The organs were harvested and used for analysis. No mouse was excluded in any group. All animals were randomly assigned (chosen alternately) to each group and not blinded. The experimental unit for the *in vivo* studies is the mouse. ALT/AST and TG levels were assessed by using Transaminase test Wako (Fujifilm) and TG test Wako (Fujifilm) in accordance with its manufacture’s protocols.

For the NASH model study (long-term miR-12135 treatment), 5-week-old male C57BL/6J mice (3 groups of 7 mice each; 21 in total) were purchased from Kyudo (Saga, Japan) for the study and acclimated for 1 week to housing conditions and an MF diet (KBT Oriental). At 6 weeks of age, the mice were randomly divided into 3 groups: Gp1 was fed an MF diet (n = 7), Gp2 was fed a CDAHFD and treated with non-targeting RNA (n = 7), and Gp3 was fed a CDAHFD and treated with miR-12135 (n = 7). Treated mice were intraperitoneally administered RNA (3 nmol/mouse) dissolved in Atelogene (Koken) (Figure 7A). On the final day, all mice were sacrificed under isoflurane vapor anesthesia. The organs were harvested and used for analyses. No mice were excluded from each group. All mice were randomly assigned (chosen alternately) to each group, and we were not blinded to this assignment. The experimental unit for our *in vivo* studies was a mouse. In the NASH model study (long-term siITGA11RNA treatment), 5-week-old male C57BL/6J mice (3 groups of 7 mice each; 21 in total) were purchased from Kyudo for the study and acclimated for 1 week to housing conditions and an MF diet (KBT Oriental). At 6 weeks of age, the mice were randomly divided into 3 groups: Gp1 was fed an MF diet (n = 7), Gp2 was fed a CDAHFD and treated with non-targeting RNA as Scr-siRNA (n = 7), and Gp3 was fed a CDAHFD and treated with siITGA11RNA (siRNA ID: 154044; Silencer, Thermo Fisher Scientific) (n = 7). Treated mice were intraperitoneally administered RNA (3 nmol/mouse) dissolved in Atelogene (Koken) (Figure 8A). On the final day, all mice were sacrificed under isoflurane vapor anesthesia. The organs were harvested and used for analyses. No mice were excluded from each group. All mice were randomly assigned (chosen alternately) to each group, and we were not blinded to this assignment. The experimental unit for our *in vivo* studies was a mouse. In the bile duct ligation (BDL) model study (miR-12135 treatment), 7-week-old male C57BL/6J mice (3 groups of 7 mice each; a total of 14 BDL-treated and 7 sham-treated mice) were purchased from Kyudo (Saga, Japan) for this study. In the BDL operation, the abdomens of mice were shaved with an electric clipper, and their epidermis was disinfected with povidone-iodine prior to making an incision with scissors in the skin and muscle layer, approximately 1 cm below the xiphoid process. The duodenum was then grasped lightly with ring tweezers, pulled out, and ligated into the upper region of the bile duct using two sutures.

Gp1 mice were sham-operated (n = 7), Gp2 mice were subjected to BDL and treated with non-targeting RNA (n = 7), and Gp3 mice were subjected to BDL and treated with miR-12135 (n = 7). Treated mice were intraperitoneally administered RNA (3 nmol/mouse) dissolved in Atelogene (Koken) (Figure 9A). BDL-treated animals were randomly assigned (chosen alternately) to each group, with equivalent average body weights maintained. We were not blinded to this assignment, and all the mice were fed an MF diet throughout the study. On the final day, all mice were sacrificed under isoflurane vapor anesthesia. The organs were harvested and used for analyses. No mice were excluded from any group, but 2 mice (both in the control group) died before sacrifice.

#### Fluorescence microscopy analysis

Harvested liver tissue was fixed by 4% PFA (Fujifilm, Tokyo, Japan) for 1 week and paraffin-embedded by Kyodo Byori (Kobe, Japan). Deparaffinization of the sections was done with Lemozol (Fujifilm, Tokyo, Japan) (for 10 min, 3 times) and EtOH (for 5 min, 3 times). Antigen retrieval was carried out using Immunosaver (Nissin EM, Tokyo, Japan) diluted by deionized water in accordance with manufacturer’s protocol and heated (95°C, 45 min). After the blocking step by 1% FBS 0.1% sodium azide containing PBS for 1 h, the section was incubated with primary antibodies (1:200 in 1% FBS 0.1% sodium azide containing PBS, overnight). After washing thrice with PBS, the section was incubated with the secondary antibody (1:1000 in 1% FBS 0.1% sodium azide containing PBS supplemented with Hoechst33342 [10000 dilution]; Thermo Fisher Scientific) for 1 h. After washing thrice with PBS, slides were treated with Vecta shield (Vector Laboratories, Burlingame, CA, USA) and observed using the BZ-X700 (Keyence) under 40× magnification. Patient samples were purchased from US BIOMAX (Cat#BC03117a). Fluorescence intensity was evaluated by ImageJ (NIH, Bethesda, MD, USA). Photo images had their brightness and contrast adjusted using PowerPoint (Microsoft, Seattle, WA).

#### Real-time PCR analysis

RNA was isolated in accordance with manufacturer’s protocol by using chloroform. The concentrations of isolated RNA were quantified using Nanodrop (Thermo Scientific) by measuring both 260 nm and 280 nm absorbance. Afterward, cDNA was obtained by using a cDNA synthesis kit (Takarabio, Shiga, Japan) and miRNA (miRCURY LNA RT Kit: 339340) in accordance with manufacturer’s protocol. The cDNA solution was assessed using RT-PCR with the Real-Time PCR System (Bio-Rad, Hercules, California) using the SYBR Premix Ex Taq (Takara Bio, Shiga, Japan) and miRNA (miRCURY LNA SYBR Green PCR Kit [4000]: 339347) following the manufacturer’s protocol. Detail information of sequence is provided as Table S3.

#### Statistical analysis

All data are shown as mean ± standard error of the mean (SEM). Significant differences between experimental variables were evaluated via Student’s t-test (two group) or Dunnett’s test using GraphPad 8.0 (GraphPad Software). p < 0.05 was considered to indicate a significant difference.

## Results

### A soy isoflavone metabolite, equol suppressed transforming growth factor β-induced fibrosis-related gene expression in LX2 cells

**Figure 1:**
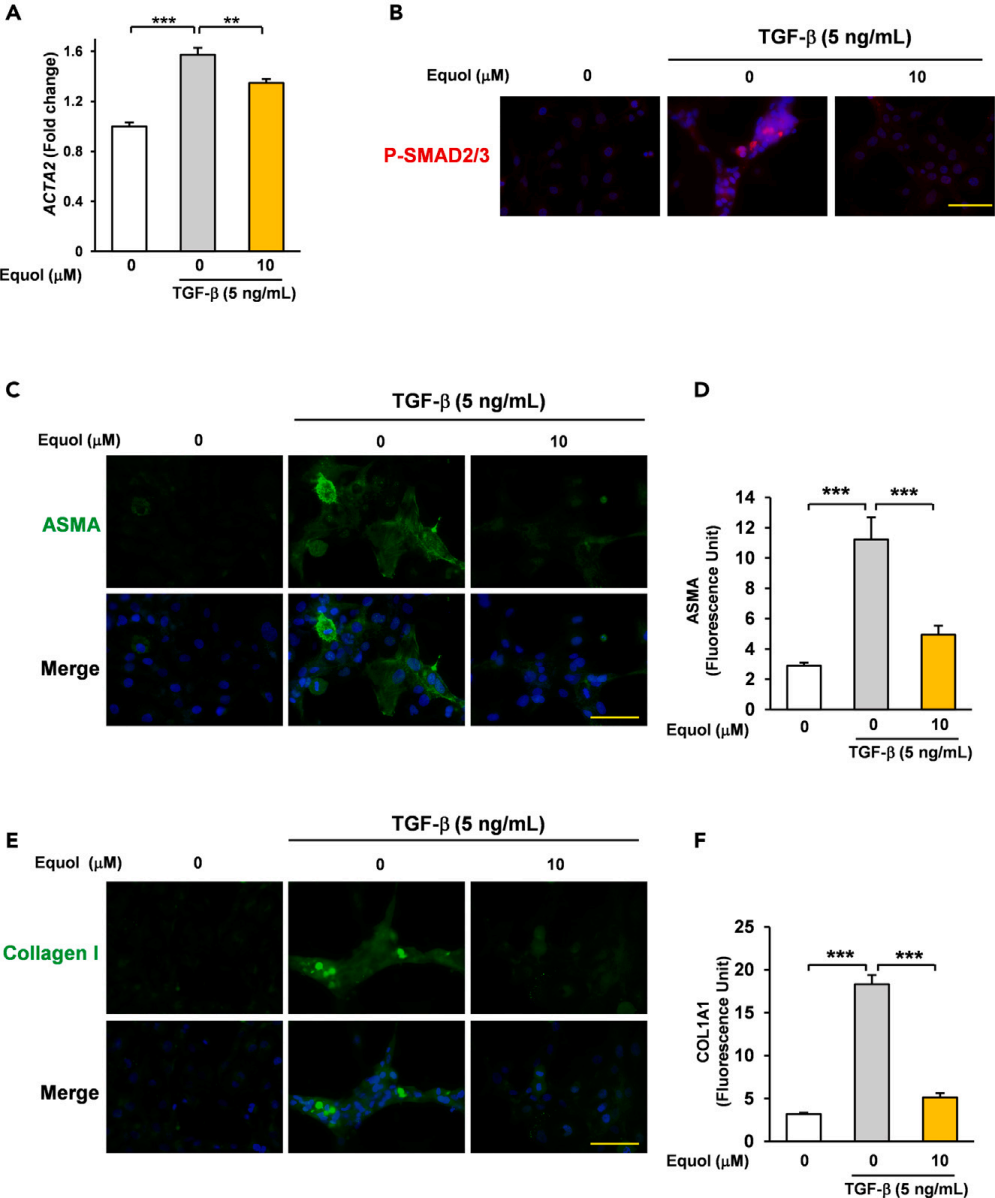
A soy isoflavone metabolite, equol suppressed TGF-β-induced fibrosis-related gene expression in LX2 cells (A) LX2 cells were treated with equol (10 μM) for 24 h and treated with TGF-β (5 ng/mL) for 48 h and assessed via qRT-PCR (n = 4). (B) LX2 cells were treated with equol (10 μM) for 24 h and with TGF-β (5 ng/mL) for 48 h, then were assessed via fluorescence microscopy. (C–F) LX2 cells were treated with equol (10 μM) for 24 h and with TGF-β (5 ng/mL) for 48 h, then were assessed via fluorescence microscopy (D) n = 34, 48, 28. (E) *n* = 50, 54, 31. The anti-fibrotic effect of equol was confirmed in 3 different experiments including this data. All data are shown as the mean ± SEM. ∗∗p < 0.01. ∗∗∗p < 0.001. (100 μm; bar).

The activation of hepatic stellate cells is a crucial step in liver fibrosis,^25^ and this is promoted by the abnormal upregulation of TGF-β secreted by other cells, including macrophages.^9^^,^^26^ Equol is a polyphenol derived from daidzein (a compound in soybeans) produced in certain microbiomes, and several studies have demonstrated its protective effects in humans.^27^^,^^28^ Previous reports revealed that soymilk showed prominent liver protective effects in human trial.^22^ Because patients with NASH with equol producing-microbiomes have relatively better status than those without,^23^ we assessed the effect of equol on the TGF-β-induced activation of human hepatic stellate cells (immortalized cell line LX-2). To achieve this, LX-2 cells were pretreated with equol (10 μM, 24 h) and treated with TGF-β (5 ng/mL, 48 h). Equol treatment suppressed the TGF-β-induced increase of ASMA levels, a well-known marker for activated hepatic stellate cells^25^ (Figure 1A).

SMAD2/3 phosphorylation is crucial step in TGF-β signaling.^29^ Translocated phosphorylated (P)- SMAD2/3 acts as a transcription factor which directly induces a variety of fibrosis-related genes.^30^ To evaluate the effect of equol on TGF-β-induced signaling in hepatic stellate cells (immortalized cell line LX-2), LX-2 cells were pretreated with equol (10 μM, 24 h) then treated with TGF-β (5 ng/mL, 48 h) and observed under fluorescence microscopy. Equol treatment was found to suppress the TGF-β-induced translocation of P-SMAD2/3 (Figures 1B and S4).

Consistent with those findings, equol treatment drastically attenuated the TGF-β-induced increases in fibrosis-related proteins such as ASMA (Figures 1C and 1D) and Collagen 1 (Figures 1E and 1F) in LX-2 cells. Differences in mRNA and protein levels would normally affect the magnitude of the difference between Acta2a and ASMA levels. In short, equol ameliorated TGF-β-induced LX2 hepatic stellate cell activation.

### A soy isoflavone metabolite, equol elicited miR-12135 upregulation

**Figure 2:**
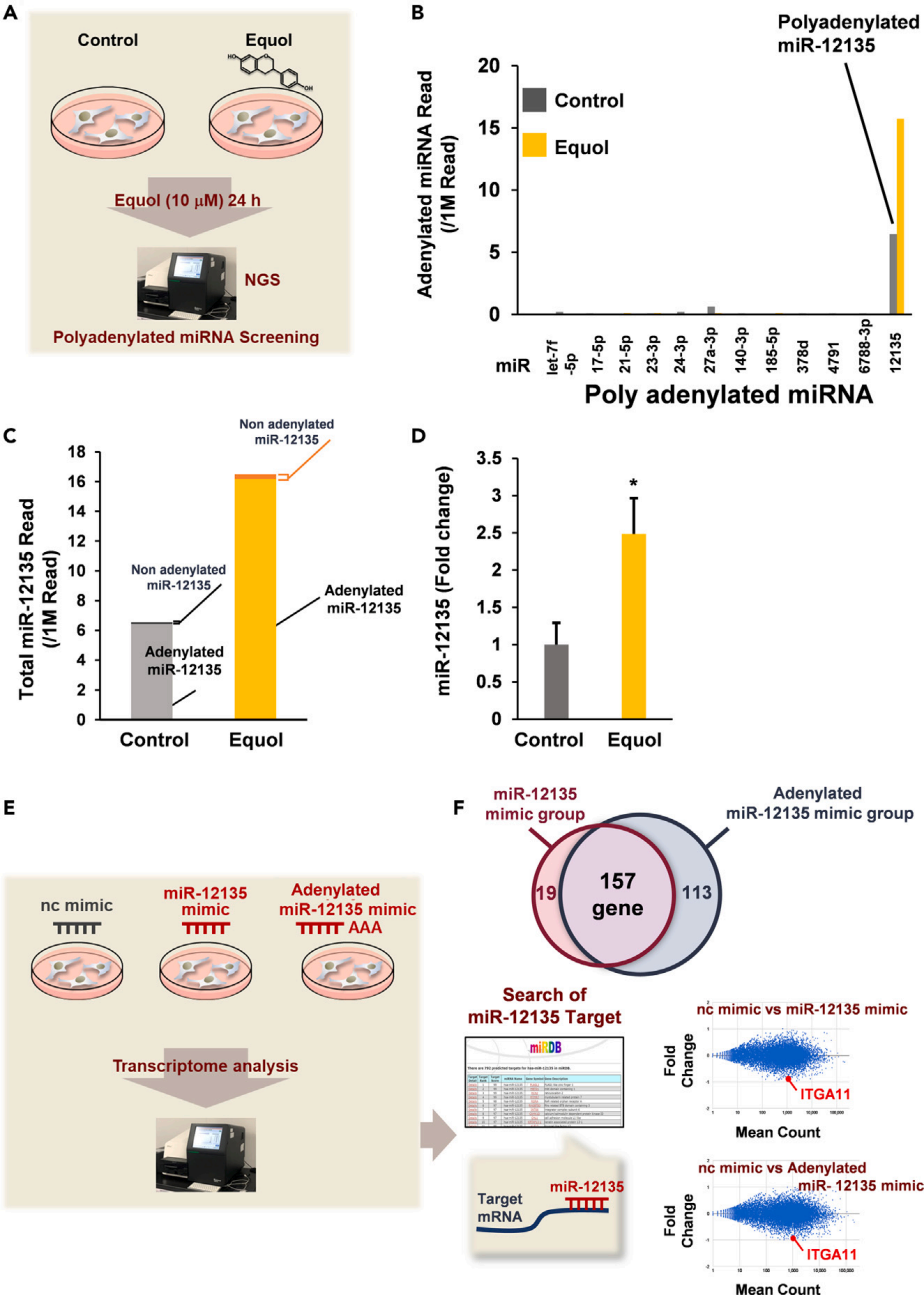
A soy isoflavone metabolite, equol elicited miR-12135 upregulation in human hepatic stellate cells (A) A scheme of the NGS experiment. (B and C) RNAseq from HeLa cells were cultured with or without 10 μM equol for 24 h. (D) Quantitative gene expression of miR-12135. Immortalized human hepatic stellate cell line LX-2 cells were treated with equol (10 μM) for 3 h and assessed via qRT-PCR (n = 4). (E and F) A scheme of the NGS experiment for identifying the target of miR-12135 (HeLa). All data are shown as the mean ± SEM. ∗p < 0.05.

Because we previously reported that PAP-associated domain containing 5 (PAPD5) mediated the pharmacological effect of equol in HeLa cells,^31^ we hypothesized that the effect of equol were mediated by the adenylation of miR because PAPD5 contains the domain that may adenylate the miRs.^32^ As we previously reported that equol increases miR adenylation levels of miR in HeLa cells via estrogen receptor-independent mechanisms^31^ and confirmed the very low levels of estrogen receptors in HeLa cells,^31^ we focused primarily on the estrogen receptor-independent effect of equol using HeLa cells and verified this by using LX2. We performed next generation sequencer (NGS) to evaluate the effect of equol on miR adenylation, and found that adenylated (A>3) miR-12135 was upregulated. (Figures 2A–2C). Consistent with those findings, equol upregulated miR-12135 expression in LX2 hepatic stellate cells (Figure 2D). Thus, equol increases miR-12135 accompanied with adenylation.

To assess the target of miR-12135 and adenylated miR-12135, we performed the RNAseq experiment followed by *in silico* analysis. HeLa cells were treated with miR-12135 mimic or adenylated miR-12135 mimic for 48 h, and transcriptome analysis was performed using an NGS. Compared with a negative control group, 176 genes were downregulated (fold change ≤0.67) by miR-12135 mimic treatment, whereas 270 genes were suppressed (fold change ≤0.67) by adenylated miR-12135 mimic. In those genes, 157 genes were commonly decreased by both miRNAs (Figures 2E and 2F). Furthermore, candidates for the target genes of miR-12135 were identified via an *in silico* analysis based on miRDB (http://mirdb.org/). We focused on ITGA11 because it acts as a collagen receptor (Figure 2F), because *in silico* analysis, miR-12135 would suppress both mouse and human ITGA11, and ITGA11 serves as the receptor for collagen (with collagen itself being the major contributor to fibrosis progression).

### miR-12135 downregulated integrin subunit alpha 11 expression in LX2 cells

**Figure 3:**
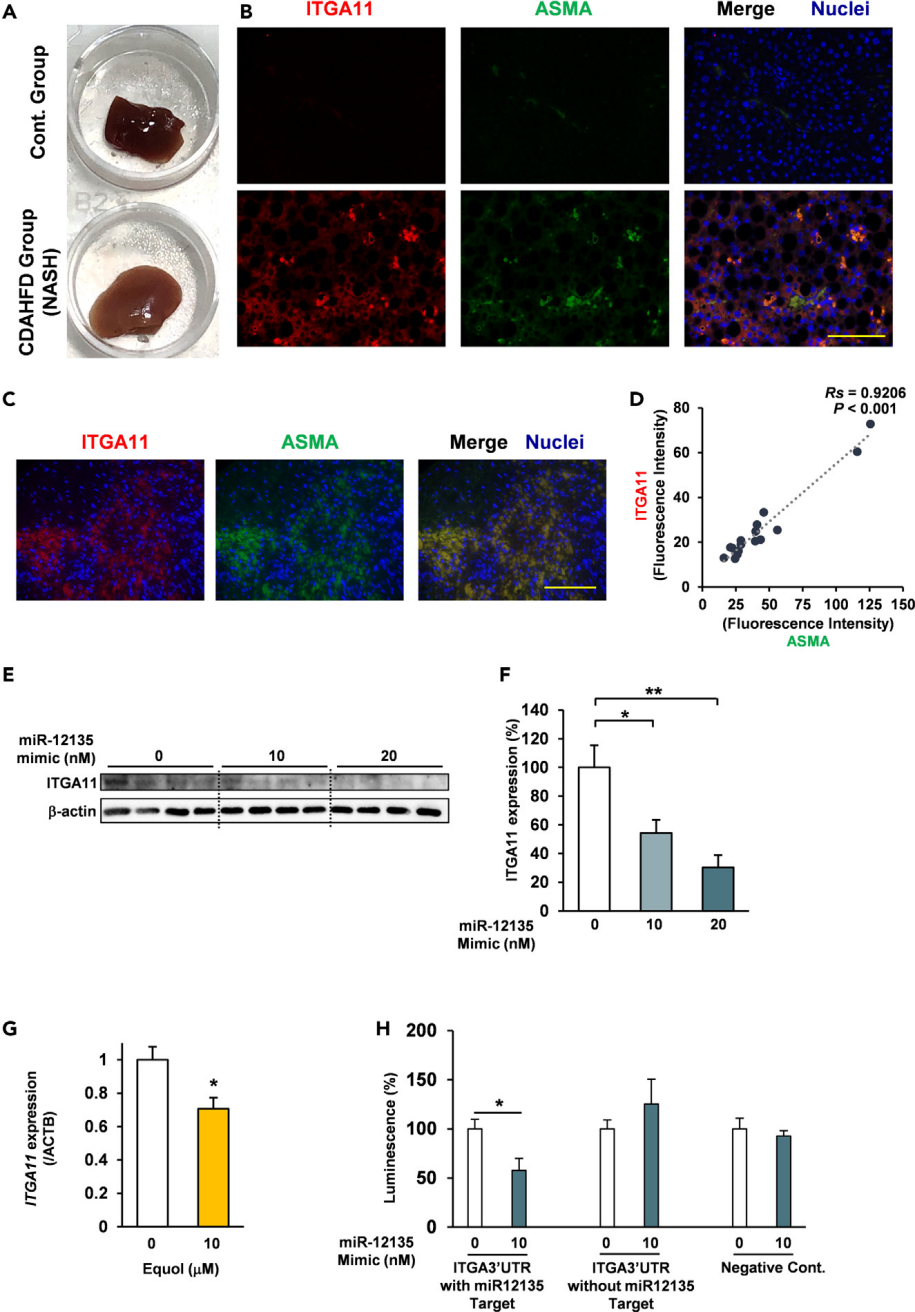
miR-12135 downregulated ITGA11 expression in human hepatic stellate cells (A) Liver tissue images of the mice fed with the MF diet and choline-deficient, L-amino acid-defined, high-fat diet (CDAHFD) (trimmed picture, whole data was shown as SIFig1A). (B) Immunofluorescence image of mouse liver tissue for ITGA11 and αSMA (marker of activated hepatic stellate cells). (C and D) Immunofluorescence image of patients with cirrhosis for ITGA11 and αSMA (marker of activated hepatic stellate cells) (n = 16). (E and F) ITGA11 (72 h) levels measured by western blotting after miR-12135 treatment in LX2 cells (n = 4). (G) LX2 cells were treated with equol (10 μM) for 24 h (n = 4). (H) Luciferase assays were carried out to assess the interaction of miR-12135 and 3′UTR of ITGA11 (n = 4). All data are shown as the mean ± SEM. ∗p < 0.05. ∗∗p < 0.01. The anti-fibrotic effect of miR-12135 was confirmed in 3 different experiments including this data. (100 μm; bar).

To assess the role of the ITGA11 in the activated hepatic stellate cells, 6-week-old male C57BL/6J mice (n = 3) were randomly entered into 2 groups and fed with the standard diet (MF det, KBT Oriental, Saga, Japan) or CDAHFD for 4 weeks, then were sacrificed under isoflurane vapor. This induced the accumulation of TG in hepatocytes and NASH followed by liver fibrosis.^33^ CDAHFD mice showed liver abnormalities (Figures 3A and S1A). Consistent with those findings, immunofluorescence staining of CDAHFD-fed mouse liver showed an increase in ASMA-positive cells, a well-known marker for activated hepatic stellate cells^25^ (Figure 3B). These findings suggest that ITGA11 is overexpressed in activated hepatic stellate cells.

Our analysis of liver tissue derived from patients with cirrhosis revealed that ITGA11 expression was significantly increased in activated hepatic stellate cells, and its expression levels correlated significantly with ASMA expression (Rs = 0.9206, n = 16, p < 0.001) (Figures 3C and 3D Patient information; Table S1). (Figures 3C and 3D Patient information; Table S1).

To confirm the effect of miR-12135 and adenylated miR-12135 on ITGA11 expression, HeLa cells were treated with miRNA and assessed via qRT-PCR (Figure S1B). Our results showed that ITGA11 was suppressed by both miR-12135 and adenylated miR-12135 (Figure S1B). We also confirmed that miR-12135 suppressed ITGA11 expression in LX2 cells in terms of protein levels in a dose-dependent manner (Figures 3E and 3F). In short, ITGA11 was suppressed by miR-12135.

LX2 cells were treated with equol (10 μM, 24 h) to assess its effect on ITGA11 expression. Equol was found to suppress ITGA11, similar to miR-12135 (Figure 3G). To confirm whether miR-12135 directly suppressed ITGA11 expression, we constructed a luciferase vector with the 3′UTR sequence changed to the 3′UTR of ITGA11. We found that miR-12135 (48 h) suppressed the luciferase activity of the cells transfected with the ITGA113′UTR sequence containing luciferase vector, but this did not affect the luciferase activity of cells transfected with negative control (NC) luciferase vector (Figure 3H). In conclusion, ITGA11 is the direct target of miR-12135.

### miR-12135 inhibited transforming growth factor β-induced fibrosis-related gene expression in LX2 cells

**Figure 4:**
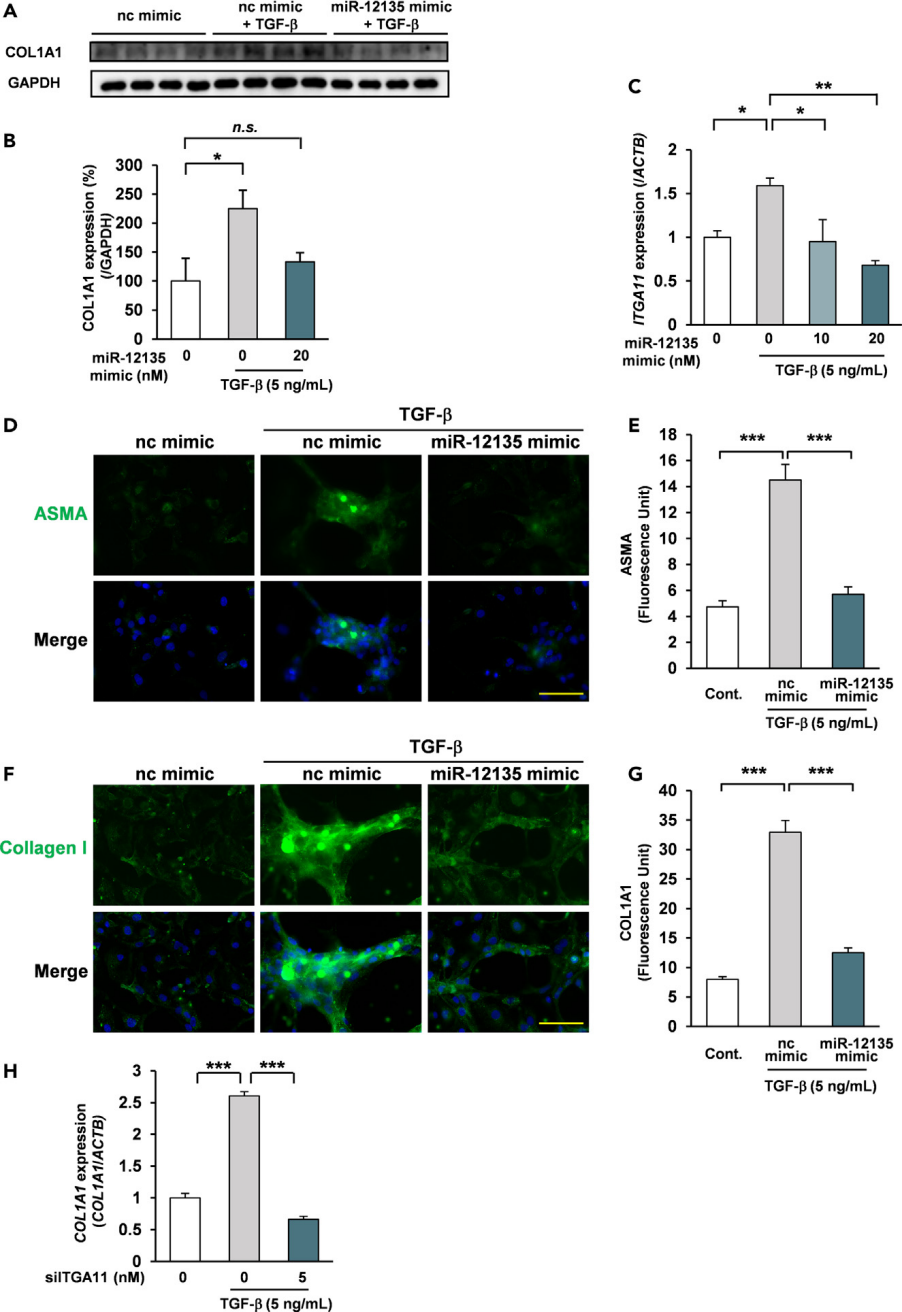
miR-12135 inhibited TGF-β-induced fibrosis-related gene expression in LX2 cells (A and B) LX2 cells were treated with miR-12135 for 24 h and with TGF-β (5 ng/mL) for 72 h, then assessed via western blotting (n = 4). (C) LX2 cells were treated with miR-12135 for 24 h and with TGF-β (5 ng/mL) for 48 h, then assessed by qRT-PCR (n = 4). (D–G) LX2 cells were treated with miR-12135 for 24 h and with TGF-β (5 ng/mL) for 48 h, then assessed by fluorescence microscopy (E) n = 27, 59, 32. (G) *n* = 32, 73, 42. (H) LX2 cells were transfected with si-ITGA11-siRNA for 24 h and with TGF-β (5 ng/mL) for 48 h, then assessed by qRT-PCR (n = 4). All data are shown as the mean ± SEM. ∗p < 0.05. ∗∗p < 0.01. The anti-fibrotic effect of siITGA11RNA was confirmed in 2 different experiments including this data. (100 μm; bar).

To assess the antifibrotic effect of miR-12135, we performed Western blot analysis of miR-12135 (20 nM, 24 h)- LX2 cells exposed to TGF-β (5 ng/mL, 72 h). Our results showed that miR-12135 completely inhibited TGF-β-induced collagen I upregulation (Figures 4A and 4B). We also assessed whether miR-12135 can sufficiently suppress ITGA11 under the existence of TGF-β in LX2 cells. Interestingly, ITGA11 was upregulated by TGF-β (5 ng/mL, 48 h), but pretreatment with miR-12135 drastically suppressed the expression level of ITGA11 (Figure 4C).

Consistent with these findings, miR-12135 pretreatment (24 h) completely neutralized TGF-β-induced expression of ASMA (Figures 4D and 4E) and Collagen I (Figures 4F and 4G). Our results showed that miR-12135 did not decrease the viable cell numbers of LX2 cells (Figure S2).

To assess whether ITGA11 downregulation is sufficient to counter TGF-β-induced fibrotic signaling, the ITGA11 of LX2 cells was treated with validated pooled siRNA against ITGA11 for reducing the off-target risk. As a result, siITGA11 treatment suppressed TGF-β-induced Collagen I expression (Figure 4H). These findings suggest that ITGA11 knocked down LX-2 cells no longer responded to TGF-β signaling. Therefore, ITGA11 is indispensable for TGF-β-induced fibrosis, while miR-12135 suppressed TGF-β-induced fibrotic signaling.

### miR-12135 ameliorated choline-deficient, L-amino acid-defined, high-fat diet-induced liver fibrosis

**Figure 5:**
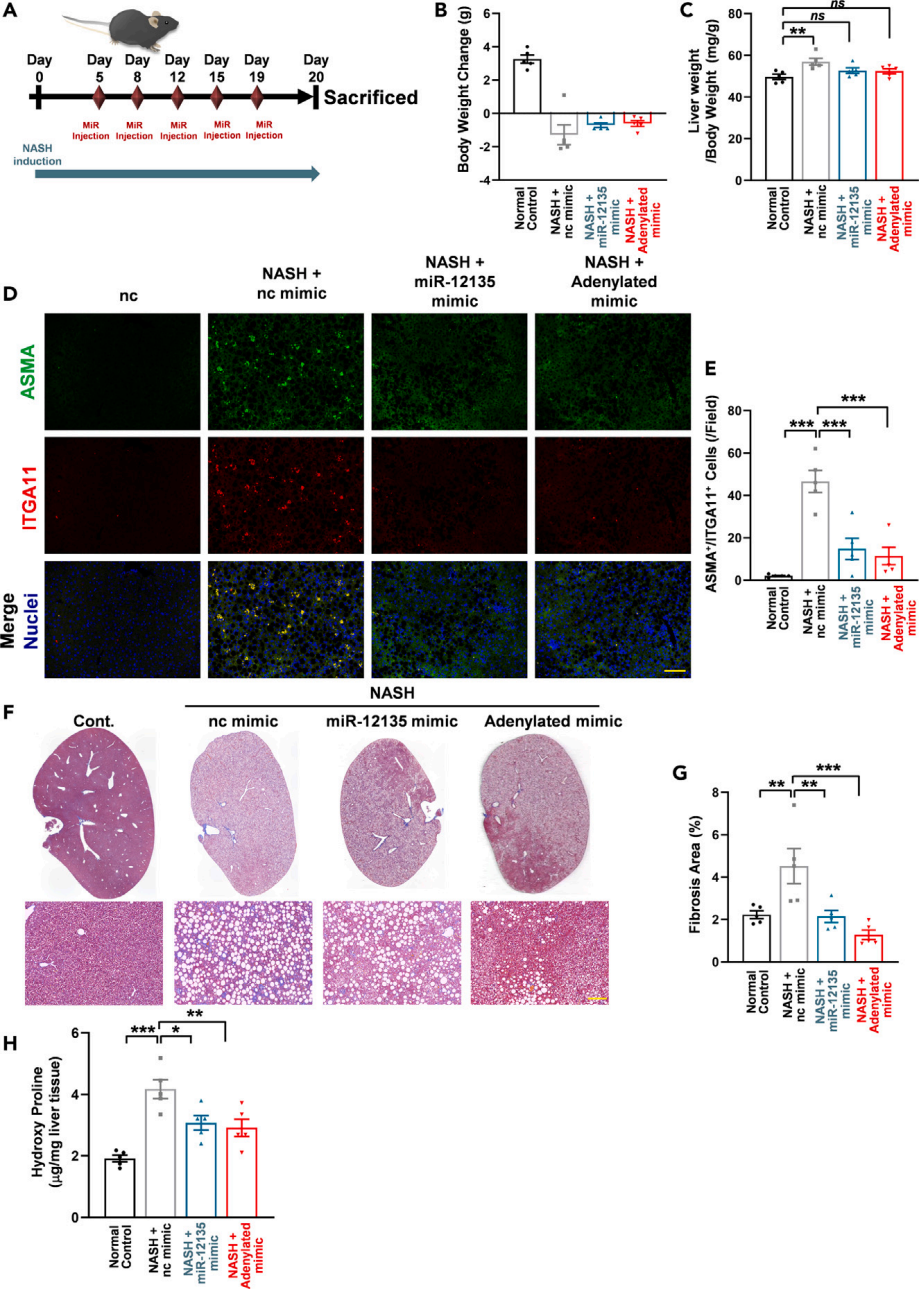
miR-12135 ameliorated CDAHFD-induced liver fibrosis (A) A scheme of the choline-deficient, L-amino acid-defined, high-fat diet (CDAHFD) mouse model. Mice are fed for 20 days with CDAHFD. (B) Mice body weight change from start to finish (day 0 to day 20) (n = 5). (C) Liver weight (mg) to body weight ratio expressed at day 20 (n = 5). (D and E) Immunofluorescence images of mouse liver tissue for ITGA11 and αSMA (marker of activated hepatic stellate cells) (n = 5). (F and G) Masson’s trichrome staining of liver tissue (n = 5). (H) Hydroxy proline levels were measured (n = 5). All data are shown as the mean ± SEM. ∗p < 0.05. ∗∗p < 0.01. ∗∗∗p < 0.001. The anti-liver fibrosis effect of miR-12135 was confirmed in 3 different mouse models including this data. (100 μm; bar).

The CDAHFD-induced NASH model is a well-established model for evaluating pharmacological effects on liver fibrosis.^33^ In this model, CDAHDF elicits the accumulation of TG in hepatocytes, which evokes several different stressors, including unfolding stress, mitochondria stress, and inflammation, causing the abnormal activation of hepatic stellate cells,^34^^,^^35^ similar to patients with cirrhosis.^36^ To assess the *in vivo* activity of miR-12135 and adenylated miR-12135 in liver fibrosis, male C57BL/6J mice (5 per group) were fed CDAHFD and mice were then given the intraperitoneal injection of miR-12135 and adenylated miR-12135 or negative control miRNA (3 nmol/mouse) dissolved in atelogene twice a week (at Days 5, 8, 12, 15, and 19). After 20 days on CDAHFD, the mice were sacrificed using isoflurane vapor and the serum and liver were harvested (Figure 5A). CDAHFD suppressed weight gain (Figure 5B), while both miR-12135 and adenylated miR-12135 slightly attenuated the CDAHFD-induced suppression of body weight gain, but this was not statistically significant (Figure 5B). An increased liver weight/body weight ratio is a well-known event in a CDAHFD-induced NASH model.^37^ CDAHFD increased the ratio in our study; however, this increase was attenuated by treatment with miR-12135 and adenylated miR-12135 (Figure 5C). Biochemical analysis showed no effect of miR-12135 on liver TG, ALT, or AST levels. (Figure S3).

To assess the effect of these miRs *in vivo*, the harvested liver tissue was evaluated via immunofluorescence chemistry analysis. Consistent with previous findings, CDAHFD upregulated ITGA11 levels in liver tissue. However, miR-12135 and adenylated miR-12135 attenuated this CDAHFD-induced ITGA11 increase (Figures 5D and 5E).

Fibrosis is a crucial prognostic factor in NASH.^38^ Masson’s trichrome (MT) staining analysis showed that both miR-12135 and adenylated miR-12135 suppressed the CDAHFD-induced liver fibrosis (Figures 5F and 5G).

Hydroxyproline is the characteristic modified amino acid in collagens.^39^ Several studies have demonstrated the suitability of hydroxyproline as a marker for the evaluation of liver fibrosis.^39^ CDAHFD increased hydroxyproline levels in the liver tissue. However, both miRs suppressed the CDAHFD-induced hydroxyproline-increase *in vivo* (Figure 5H). In conclusion, miR-12135 and adenylated miR-12135 showed strong antifibrotic effects accompanied with the suppression of ITGA11 in a CDAHFD-induced NASH model.

### miR-12135 attenuated transforming growth factor β signaling in hepatic stellate cells

**Figure 6:**
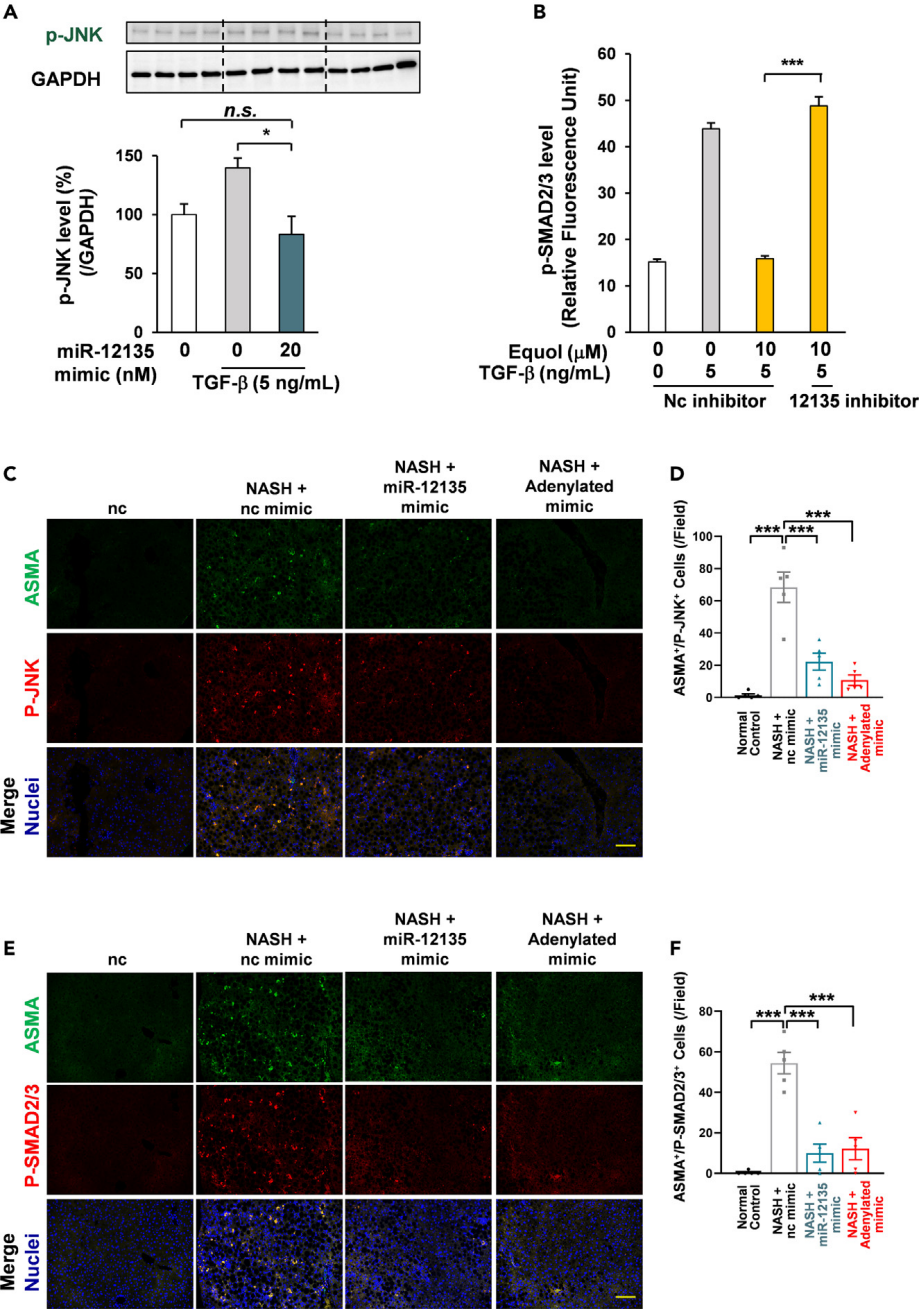
miR-12135 attenuated TGF-β signaling in hepatic stellate cells (A) LX2 cells were treated with miR-12135 for 24 h and with TGF-β (5 ng/mL) for 0.5 h (n = 4) Tukey’s test. (B) LX2 cells were treated with miR-12135 (10 nM) inhibitor for 6 h, pretreated with equol (10 μM), and treated with TGF-β (5 ng/mL) for 48 h (n = 86, 133, 36, 111). (C and D) Immunofluorescence images of mouse liver tissue of Figure 5 for P-JNK and αSMA (marker of activated hepatic stellate cells) (n = 5). (E and F) Immunofluorescence images of mouse liver tissue of Figure 5 for P-SMAD2/3 and αSMA (marker of activated hepatic stellate cells) (n = 5 mice). All data are shown as the mean ± SEM. ∗p < 0.05. ∗∗∗p < 0.001. The attenuating effect of miR-12135 inhibitor on the anti-fibrotic effect of equol was confirmed in multiple experiments including this data. The suppressive effect of miR-12135 on P-SMAD2/3 was confirmed in 3 different mouse models including this data. (100 μm; bar).

In a previous study, it has been reported that ITGA11 regulated JNK phosphorylation in cancer-associated fibroblasts.^40^ Furthermore, P-JNK is involved in the phosphorylation of SMAD2/3.^41^ We hypothesized that miR-12135 inhibited TGF-β signaling by suppressing P-JNK and P-SMAD2/3 levels through the downregulation of ITGA11. To assess the effect of miR-12135 on the phosphorylation of JNK and SMAD2/3, LX2 cell were pretreated with miR-12135 mimic then treated with TGF-β. Western blot analysis showed that miR-12135 suppressed the level of P-JNK induced by TGF-β (Figure 6A). To evaluate the role of miR-12135 in the antifibrotic effect of equol, human LX-2 cells were pretreated with miR-12135 inhibitor then treated with equol. Equol treatment suppressed the TGF-β-induced upregulation of P-Smad2/3 levels (Figure 6B) and its levels in nuclei (Figure S4). In contrast, miR-12135 inhibitor treatment canceled the antifibrotic effect of equol (Figures 6B and S6). In summary, miR-12135 was involved in the antifibrotic effect of equol. To evaluate the effect of miR-12135 in CDAHFD-fed mice, P-JNK and P-Smad2/3 levels were assessed by using immunofluorescence analysis. This revealed that treatment with miR-12135 mimic and adenylated miR-12135 mimic canceled the upregulation of P-JNK and P-Smad2/3 levels (Figures 6C–6F). In summary, equol suppressed TGF-β signaling by the upregulation of miR-12135. Furthermore, miR-12135 suppressed ITGA11 and the CDAHFD-induced upregulation of P-JNK and P-Smad2/3 levels.

### miR-12135 ameliorated choline-deficient, L-amino acid-defined, high-fat diet-induced liver fibrosis for long term model

**Figure 7:**
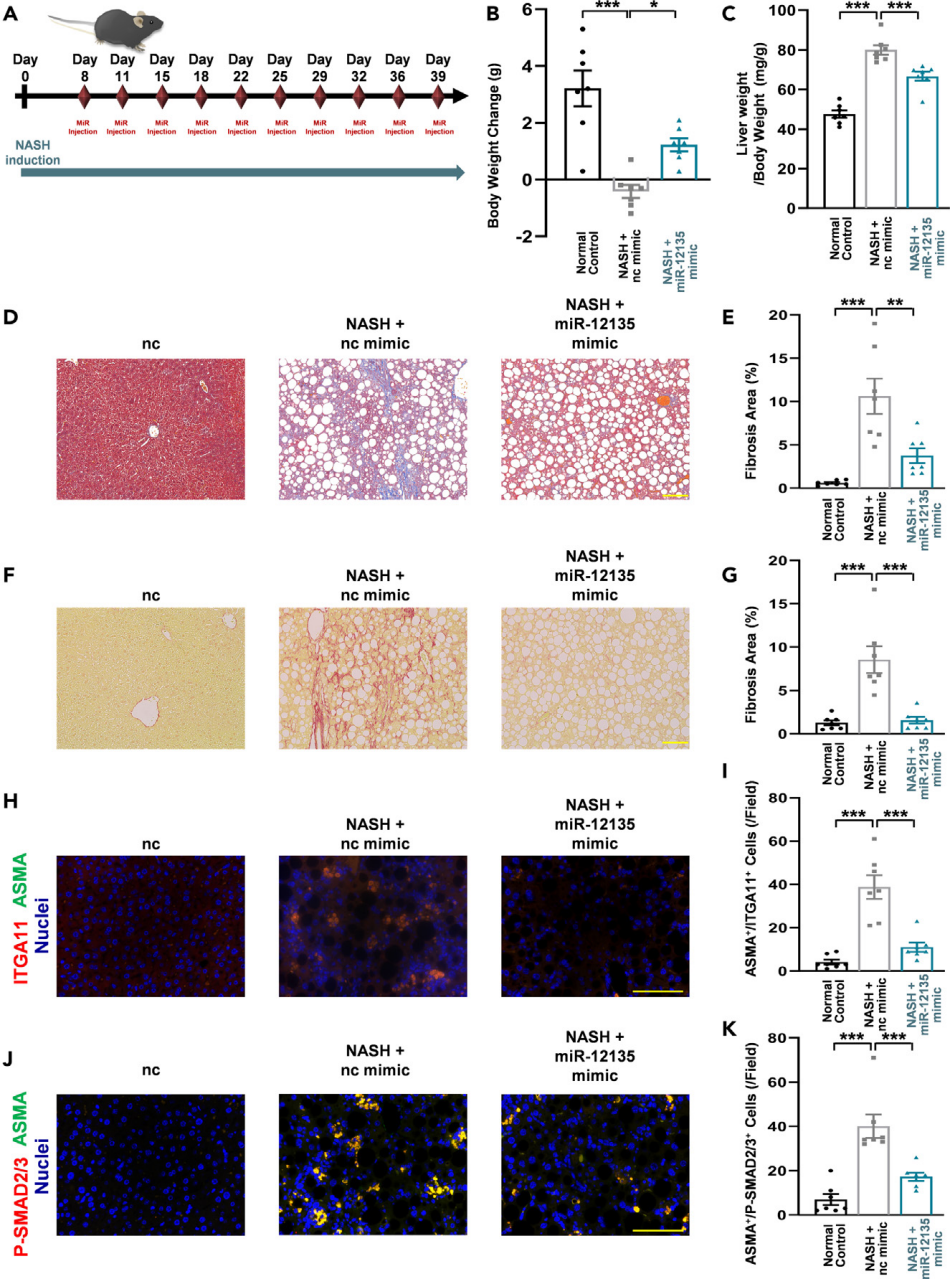
miR-12135 ameliorated CDAHFD-induced liver fibrosis for long term model (A) A scheme of the choline-deficient, L-amino acid-defined, high-fat diet (CDAHFD) mouse model. Mice are fed for 40 days with CDAHFD. (B) Mice body weight change from start to finish (day 0 to day 40) (n = 7). (C) Liver weight (mg) to body weight ratio expressed at day 40 (n = 7). (D and E) Masson’s trichrome staining of liver tissue (n = 7). (F and G) Picro-Sirius Red staining of liver tissue (n = 7). (H and I) Immunofluorescence images of mouse liver tissue for ITGA11 and αSMA (marker of activated hepatic stellate cells) (n = 7). (J and K) Immunofluorescence images of mouse liver tissue for P-SMAD2/3 and αSMA (marker of activated hepatic stellate cells) (n = 7). All data are shown as the mean ± SEM. ∗p < 0.05. ∗∗p < 0.01. ∗∗∗p < 0.001. The anti-liver fibrosis effect of miR-12135 was confirmed in 3 different mouse models including this data. (100 μm; bar).

To assess the long-term effect of miR-12135 on liver fibrosis, male C57BL/6J mice (7 per group) were fed a CDAHFD for 6 weeks with an intraperitoneal injection of miR-12135 (3 nmol/mouse) dissolved in Atelogene (Figure 7A). After 40 days on a CDAHFD, the mice were sacrificed using isoflurane vapor anesthesia, and their serum and livers were harvested (Figure 7A). CDAHFD suppressed weight gain (Figure 7B), whereas miR-12135 attenuated CDAHFD-induced suppression of body weight gain (Figure 7B). An increased liver weight/body weight ratio is a well-known event in CDAHFD-induced NASH models.^37^ CDAHFD increased this ratio in our study. This increase was attenuated by miR-12135 treatment (Figure 7C). Biochemical analysis showed no effect of miR-12135 on ALT/AST levels (Figure S5). Fibrosis is a crucial prognostic factor for NASH.^38^ MT staining analysis showed that miR-12135 suppressed CDAHFD-induced liver fibrosis (Figures 7D and 7E). Consistent with the MT staining analysis, Picro-Sirius Red staining also demonstrated that miR-12135 suppressed CDAHFD-induced liver fibrosis (Figures 7F and 7G).

To assess the effect of miR *in vivo*, harvested liver tissues were evaluated using immunofluorescence. Consistent with previous findings, CDAHFD upregulated ITGA11 and P-SMAD2/3 levels in liver tissue, whereas miR-12135 attenuated CDAHFD-induced ITGA11 (Figures 7H and 7I) and P-SMAD2/3 (Figures 7J and 7K) increases. In conclusion, miR-12135 showed significant efficacy in two different CDAHFD-fed mice models (Figures 5 and 7).

### siITGA11 RNA treatment ameliorated choline-deficient, L-amino acid-defined, high-fat diet-induced liver fibrosis for long term model

**Figure 8:**
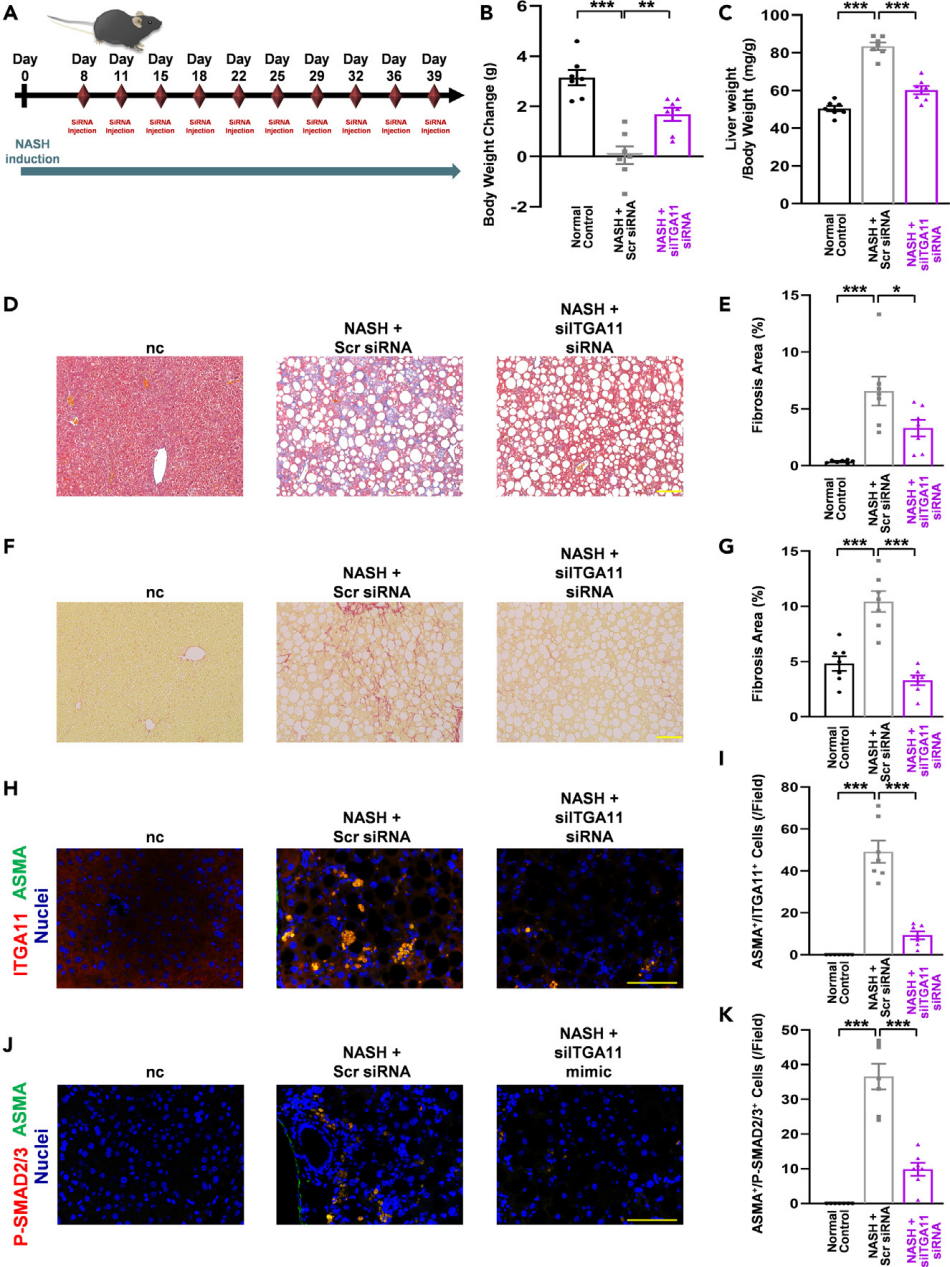
siITGA11 RNA treatment ameliorated CDAHFD-induced liver fibrosis for long term model (A) A scheme of the choline-deficient, L-amino acid-defined, high-fat diet (CDAHFD) mouse model. Mice are fed for 40 days with CDAHFD. (B) Mice body weight change from start to finish (day 0 to day 40) (n = 7). (C) Liver weight (mg) to body weight ratio expressed at day 40 (n = 7). (D and E) Masson’s trichrome staining of liver tissue (n = 7). (F and G) Picro-Sirius Red staining of liver tissue (n = 7). (H and I) Immunofluorescence images of mouse liver tissue for ITGA11 and αSMA (marker of activated hepatic stellate cells) (n = 7). (J and K) Immunofluorescence images of mouse liver tissue for P-SMAD2/3 and αSMA (marker of activated hepatic stellate cells) (n = 7). All data are shown as the mean ± SEM. ∗p < 0.05. ∗∗p < 0.01. ∗∗∗p < 0.001. (100 μm; bar).

To evaluate the long-term impact of siITGA11RNA treatment on liver fibrosis, male C57BL/6J mice (7 per group) were fed a CDAHFD for 6 weeks and administered intraperitoneal injections of siITGA11RNA or non targeting RNA (3 nmol/mouse) dissolved in Atelogene (Figure 8A). After 40 days on a CDAHFD, the mice were sacrificed using isoflurane vapor anesthesia, and their serum and livers were harvested (Figure 8A). CDAHFD suppressed weight gain (Figure 8B) and siITGA11RNA attenuated this CDAHFD-induced suppression of body weight gain (Figure 8B). An increased liver weight/body weight ratio is a well-known event in CDAHFD-induced NASH models.^37^ CDAHFD increased this ratio in our study, and this increase was attenuated by siITGA11RNA treatment (Figure 8C). Biochemical analysis revealed no effect of siITGA11RNA on ALT/AST levels (Figure S6). Fibrosis is a crucial prognostic factor for NASH.^38^ MT staining analysis showed that siITGA11RNA suppressed CDAHFD-induced liver fibrosis (Figures 8D and 8E). Consistent with the MT staining analysis, Picro-Sirius Red staining demonstrated that siITGA11RNA suppressed CDAHFD-induced liver fibrosis (Figures 8F and 8G).

To assess the role of ITGA11 *in vivo*, we evaluated harvested liver tissues using immunofluorescence. Consistent with previous findings, CDAHFD upregulated ITGA11 and P-SMAD2/3 levels in liver tissues, and siITGA11RNA attenuated CDAHFD-induced ITGA11 (Figures 8H and 8I) and P-SMAD2/3 (Figures 8J and 8K) upregulation. In conclusion, knockdown of miR-12135 target ITGA11 significantly suppressed CDAHFD-induced liver fibrosis in this mouse model (Figure 8).

### miR-12135 ameliorated bile duct ligation-induced liver fibrosis

**Figure 9:**
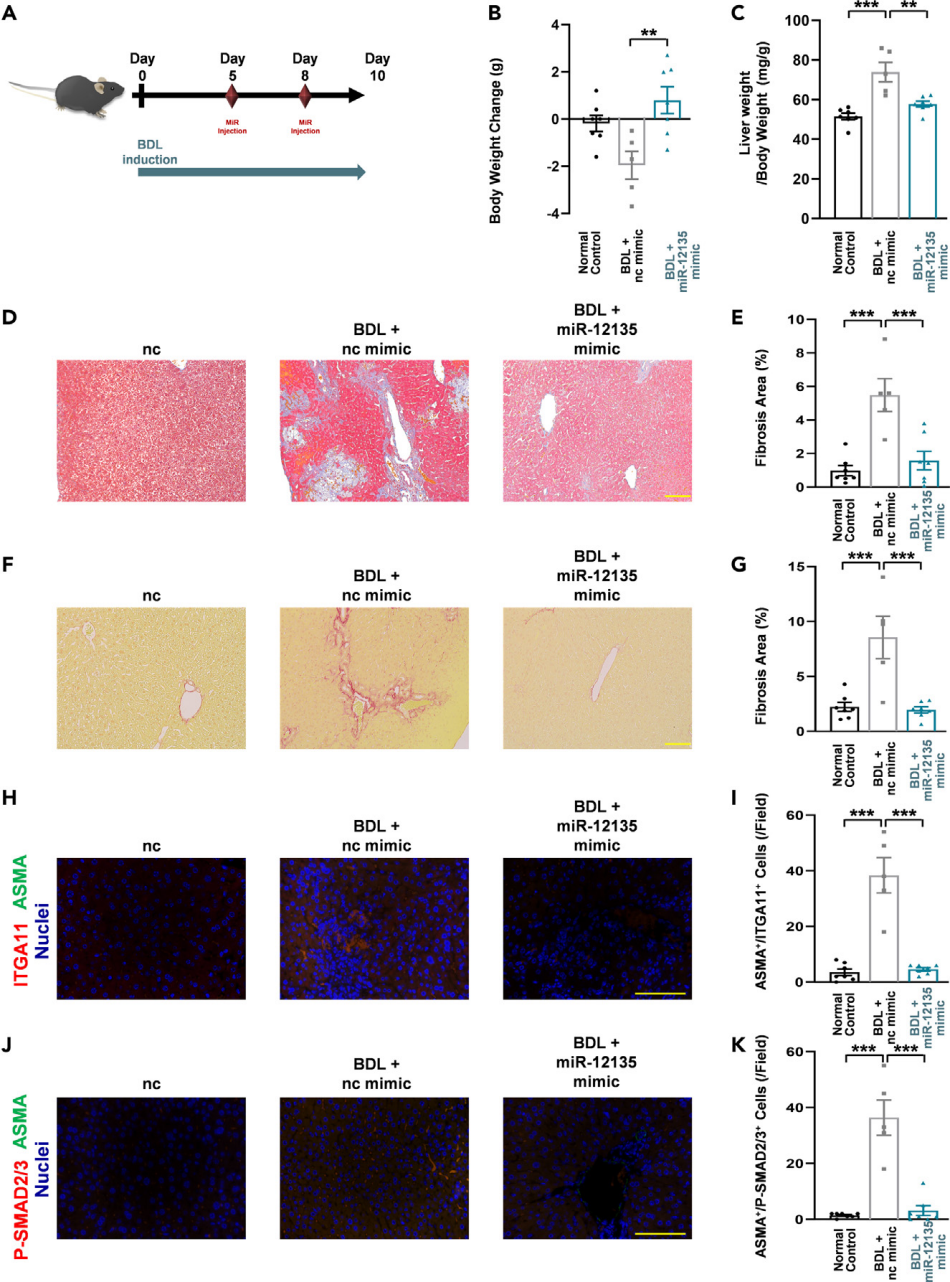
miR-12135 ameliorated BDL-induced liver fibrosis (A) A scheme of the bile duct ligation (BDL)-induced liver fibrosis mouse model (all group were started as (n = 7) and 2 mice (control group) were dead before sacrifice and finally (n = 7, 5, 7). (B) Mice body weight change from start to finish (treatment start to the final) (n = 7, 5, 7). (C) Liver weight (mg) to body weight ratio expressed at the end (n = 7, 5, 7). (D and E) Masson’s trichrome staining of liver tissue (n = 7, 5, 7). (F and G) Picro-Sirius Red staining of liver tissue (n = 7, 5, 7). (H and I) Immunofluorescence images of mouse liver tissue for ITGA11 and αSMA (marker of activated hepatic stellate cells) (n = 7, 5, 7). (J and K) Immunofluorescence images of mouse liver tissue for P-SMAD2/3 and αSMA (marker of activated hepatic stellate cells) (n = 7, 5, 7). All data are shown as the mean ± SEM. ∗p < 0.05. ∗∗p < 0.01. ∗∗∗p < 0.001. The anti-liver fibrosis effect of miR-12135 was confirmed in 3 different mouse models including this data. (100 μm; bar).

BDL is a major disease model for liver fibrosis.^42^ To assess the effect of miR-12135 on liver fibrosis under normal diet conditions, male C57BL/6J mice (7 per group) were BDL-operated (NC group mice (7 mice/group) were sham-operated). Mice were then administered intraperitoneal injections of miR-12135 or negative control miRNA (3 nmol/mouse) dissolved in Atelogene (Figure 9A). 10 days after the BDL treatment, the mice were sacrificed using isoflurane vapor anesthesia, and their serum and livers were harvested (Figure 9A). During this study, two mice (ncmiR mimic treated-BDL mice) died before sacrifice. miR-12135 increased body weight gain compared to ncmiR mimic treated-BDL mice (Figure 9B). BDL treatment increased the liver/BW ratio in our study, and this increase was attenuated by miR-12135 treatment (Figure 9C). Biochemical analysis revealed that miR-12135 treatment suppressed ALT levels (Figure S7). MT staining analysis showed that miR-12135 suppressed BDL-induced liver fibrosis (Figures 9D and 9E). Consistent with the MT staining analysis, Picro-Sirius Red staining also demonstrated that miR-12135 suppressed BDL-induced liver fibrosis (Figures 9F and 9G).

To assess the *in vivo* effect of miR in the BDL model, harvested liver tissues were evaluated using immunofluorescence. BDL upregulated ITGA11 and P-SMAD2/3 levels in liver tissues (Figures 9H–9K). Consistent with CDAHFD-induced liver fibrosis, miR-12135 attenuated BDL-induced ITGA11 (Figures 9H and 9I) and P-SMAD2/3 (Figures 9J and 9K) increases. Taken together, miR-12135 showed significant efficacy in our BDL-induced liver fibrosis model and in both different term CDAHFD-fed mouse models (Figures 5, 7, and 9).

## Discussion

Several processes are involved in the progression of NASH, including obesity-induced chronic inflammation, mitochondrial damage elicited by TG accumulation, and ER stress.^3^^,^^4^^,^^5^^,^^6^^,^^7^ These factors induce hepatocyte cell death and damage associated molecular patterns (DAMPs), which enhance inflammation.^9^ In the process of liver fibrosis, macrophages produce TGF-β which triggers the activation of hepatic stellate cells followed by collagen overproduction.^9^ Fibrosis an important independent factor that is positively correlated with the risk of hepatic cancer.^11^ However, current therapeutic strategies focus on the improvement of glucose intolerance and few of the current approaches aim to improve the abnormal activation of hepatic stellate cells and fibrosis, and thus a novel strategy is now strongly demanded. PPAR-γ is involved in HSC activation and thiazolidinediones affect fibrosis. Several guidelines recommend TZDs for treating patients with NASH.^43^ TGF-β production induced by inflammatory cytokines from several types of cells plays a crucial role in hepatic fibrosis.^9^ Accordingly, several *in vitro* and *in vivo* studies have indicated that TGF-β-induced SMAD2/3 activation is a central mechanism in fibrosis.^29^^,^^30^ However, this signaling pathway is also indispensable in several physiological process (e.g., immune tolerance),^12^ and thus it is difficult to target this axis with the goal of suppressing fibrosis.

Based on NGS analysis followed by *in silico* analysis for target prediction, we revealed that functionally unknown miR-12135 induced by equol, a metabolite of the soybean polyphenol daidzein, drastically suppressed the fibrosis-related genes in hepatic stellate cells by ITGA11 suppression. In a study on CDAHFD-treated mice, we also confirmed that miR-12135 treatment neutralized CDAHFD-induced fibrosis and suppressed ITGA11 in the activated hepatic stellate cells. Moreover, we confirmed that ITGA11 is colocalized with ASMA in those patients‘ tissue. Consistent with those findings, ITGA11 knockdown in hepatic stellate cells significantly compromised the TGF-β-induced fibrosis signaling. This data indicated that TGF-β-induced ITGA11 upregulation is an indispensable mediator for TGF-β signaling. Considering the significant correlation between the expressions of ITGA11 and ASMA (the major severity marker for liver fibrosis),^25^ a positive feedback loop may contribute to the excess activation of hepatic stellate cells. As ASMA is the marker for activated stellate cells, the coexistence of ITGA11 in activated stellate cells is to be expected because we found that ITGA11 is induced by TGFβ. However, although ITGA11 is upregulated by TGFβ, our results showed that ITGA11 is essential for TGFβ-induced upregulation of Col1a. We also found that miR-12135 suppressed ITGA11 expression and ASMA levels. Owing to the complicated relationships between these phenomena, it is impossible to elucidate a ‘chicken-and-egg’ relationship.

Previous studies have suggested the involvement of ITGA11 in JNK phosphorylation, alongside the direct interaction of JNK.^40^ Consistent with these reports, miR-12135 treatment significantly canceled CDAHFD-induced P-JNK-positive activated hepatic stellate cells. Moreover, miR-12135 treatment also suppressed the TGF-β-induced increase of P-JNK levels. These findings indicate that miR-12135 downregulated P-JNK levels by suppressing the expression of ITGA11.

Notably, ITGA11 is upregulated in activated hepatic stellate cells, whereas ITGA11 knockout mice are viable and fertile.^24^ Thus, ITGA11 itself could be a novel therapeutic target for patients with NASH. Our results showed that ITGA11 knockdown strongly suppressed liver fibrosis in the CDAHFD model. Recently, by using hepatic stellate cells that selectively intake all-*trans* retionic acid (ATRA), drug delivery of siRNA to hepatic stellate cells has been established.^44^ Clinical trials are currently using hepatic stellate cell-targeting vesicles which contain siRNA for heat shock protein 47 (HSP47), the central component for collagen production.^45^ Considering the role of overactivated hepatic stellate cells in NASH, ITGA11 is a potential target because it overexpressed in the activated hepatic stellate cells and is indispensable in their activation. Taking these into consideration, miR-12135 may provide efficient countermeasures that could fundamentally reverse NASH, as demonstrated in CDAHFD-fed mice treated with miR-12135 under two different conditions and in a BDL-induced liver fibrosis model. Moreover, considering the mechanism of the anti-fibrotic effects of miR12135 is based on novel mechanisms, the additional effect on the conventional NASH treatment (anti-diabetic drug) could be expected.

A human clinical trial showed the potent effect of soymilk in NASH.^22^ Considering the drastic effect of miR12135 in mice, the protective effect of equol itself is promising. However, a limitation of this study is that we could not evaluate the role of miR-12135 in equol-treated mice because these mice did not have the miR-12135 gene.

Most of the miR-12135 produced in response to equol was polyadenylated. Considering the minor differences in efficacy and target genes between the miR-12135 mimic and polyadenylated miR-12135, adenylation is expected to stabilize miR-12135. However, in the *in vivo* experiments, we administered miRs with the stabilizer Atelogene, which masked the difference. As previously reported, 160 mg equol BID results in a −10 μM plasma equol concentration.^46^ In rats, the equol concentration resulting from a 6 μM oral dose represents the daily intake of a 200 mg racemic equol/kg diet, which is a physiologically relevant level for dietary racemic equol.^47^

A previous report indicates that the beneficial effect of soy milk in NASH.^22^ Equol, a metabolite derived from daidzein is one of the active compounds derived from soy milk.^27^^,^^28^ However, little is known about its efficacy and mechanisms. Here we showed that miR-12135 mediated the anti-fibrotic effect of equol in the TGF-induced fibrosis signaling and to my knowledge, it is the first report by miRDB analysis.^48^

In conclusion, equol upregulated miR-12135 and showed antifibrotic effects. Moreover, miR-12135 downregulated ITGA11 and suppressed the TGF-β- or CDAHFD-induced upregulation of P-JNK and P-SMAD2/3 levels. Therefore, the miR-12135/ITGA11 axis could be a novel therapeutic target in NASH.

### Limitations of the study

Limitation of this study is that we could not evaluate the role of miR-12135 in equol-treated mice because these mice did not have the miR-12135 gene. Also, considering the minor differences in efficacy and target genes between the miR-12135 mimic and polyadenylated miR-12135, adenylation is expected to stabilize miR-12135. However, in the *in vivo* experiments, we administered miRs with the stabilizer Atelogene, which could mask the difference.

## STAR★Methods

### Key resources table

**Table:** 

REAGENT or RESOURCE	SOURCE	IDENTIFIER
**Antibodies**		

Anti-P-SMAD2/3 Ab	Cell Signaling Technology	#8828S
Anti-ASMA Ab	Cell Signaling Technology	#48938S
Anti-Collagen1 Ab	Santa Cruz	sc-59772
Anti-ITGA11 Ab	Abcam	ab198826
Anti-P-JNK Ab	R&D Systems	AF1205-SP
Anti-beta act-Ab	Sigma Aldrich	A2228
Anti-GAPDH Ab	Cell Signaling Technology	#5174S
Anti-mouse AF488 Ab	Thermo Fisher Scientific	A11017
Anti-Rabbit AF555 Ab	Thermo Fisher Scientific	A21428

**Biological samples**		

CDAHFD	Research Diet	A06071302
Liver Tissue array	US bio max	Cat#BC03117a

**Chemicals, peptides, and recombinant proteins**		

Equol	Tokyo Chemical Industry	E0922
HOECHST 33342	Thermo Fisher Scientific	H3570
PBS	Fujifilm	049-29793
DMEM	Fujifilm	044-29765
Streptomycin	Meiji Pharmaceutical Co.	876161
Penicillin G	Meiji Pharmaceutical Co.	876111
Fetal bovine serum	Sigma Aldrich	172012
FuGENE®6 Transfection Reagent	Promega	REF:E269A
ONE-Glo™ EX Luciferase Assay Substrate	Promega	REF:E633A
Optimem	Thermofisher	31985-062
Tripsin EDTA	Fujifilm	208-17251
Lemosol	Fujiflm	128-03993
Mounting meddium	Vector Laboratories	H-1000
4% Paraformaldehyde	Fujifilm	163-20145
TGF beta	R and D systems	100-B-001
Trans Blot nitrocellulose membranes	Sigma Aldrich	Protran BA 85
TMA-6	Lumigen	TMA-6
NGS equipment	Nova Seq	N/A
Trireagent	Cosmo Bio	TR118
Lipofectamine RNAiMAX Transfection Reagent	Thermo Fisher Scientific	13778150
Attelogene	Koken	1391

**Critical commercial assays**		

ATPlite	PerkinElmer	6016731
Histology staining	Kyodo Byori	N/A
QuantiTect Rev. Transcription Kit (400)	Qiagen	205314
PowerUp SYBR Green Master Mix	Qiagen	A25776
ALT/AST analysis kit	Fujifilm	431-30901
TG kit	Fujifilm	432-40201
Hydroxyproline analysis kit	Abcam	ab222941

**Experimental models: Cell lines**		

LX2	Merck	N/A
HeLa	ATCC	N/A

**Experimental models: Organisms/strains**		

C57BL6J mouse	Kyudo	N/A

**Oligonucleotides**		

Control Vector	GeneCopoeia	CMIT000001-MT06
miR12135 primer	Qiagen	YCP0044878
primers for mRNA analysis	Eurofins genomics	(SI Tables)
ITGA3’UTR with miR12135 Target Vector	GeneCopoeia	HmiT130500-MT06
ITGA3’UTR without miR12135 Target Vector	GeneCopoeia	HmiT130500-MT06
miR12135	Sigma Aldrich	N/A
siITGA11	Dharmacon	siRNAID:154044
polyA miR12135	Sigma Aldrich	N/A
miR12135 inhibitor	Ambion	Cat#4464084

**Software and algorithms**		

Powerpoint 2019	Microsoft	N/A
Excel 2019	Microsoft	N/A
Graphpad Prism 8.0	GraphPad Software	N/A
Image J	NIH	N/A
Kyplot 6.0	KyensLab Inc	N/A

**Other**		

Plate reader Envision™™	PerkinElmer	Envision™
Kyence BZ-X700	Keyence	BZ-X700™
WB detector Fusion System	Vilber-Lourmat	Fusion System™
CFX 96Real-Time PCR System	Bio-Rad	N/A
Hi seq	Illumina	N/A

### Resource availability

#### Lead contact

Dr. Hirofumi Tachibana.

Division of Applied Biological Chemistry, Department of Bioscience and Biotechnology, Faculty of Agriculture, Kyushu University, Japan.

Email: tatibana@agr.kyushu-u.ac.jp.

#### Materials availability

This study did not generate new reagents.

#### Data and code availability

The data presented in this paper, it can be obtained by lead contact tatibana@agr.kyushu-u.ac.jp.

This paper does not generate original code.

### Method details

#### Liver fibrosis model (CDAHFD)

Because the pharmacological effects of miR-12135 were not predicted, group size was not calculated. All mice used in this study were maintained in an approximately humid (60%), temperature-controlled (20°C) room with a 12-h light–dark cycle (dark from 8 PM to 8 AM). Mice were allowed *ad libitum* accessed to diet and drinking water. We confirm that the study is in accordance with the ARRIVE guidelines.

In the NASH model (immunohistochemistry staining in Figure 3), 5-week-old male C57BL/6J mice (2 groups, 3 mice each) were purchased from Kyudo (Saga, Japan) and acclimated for 1 week by being fed MF diet (KBT Oriental, Saga, Japan).

At 6 weeks of age, mice were randomly entered into 2 groups: Gp1 was fed with MF diet (n = 3), while Gp2 was fed with CDAHFD (Research Diet, A06071302) (n = 3). After 4 weeks, all mice were sacrificed under isoflurane vapor. The organs were harvested and used for analysis. No mouse was excluded in any group.

In the NASH model (miR-12135 treatment), 5-week-old male C57BL/6J mice (4 groups, 5 mice each; total of 20) were purchased from Kyudo (Saga, Japan) and acclimated for 1 week by being fed MF diet (Saga, Japan).

At 6 weeks of age, mice were randomly entered into 4 groups: Gp1 was fed with MF diet (n = 5), Gp2 was fed with CDAHFD and not treated with targeting miRNA (n = 5), Gp3 was fed with CDAHFD and treated with miR-12135 (n = 5), Gp4 was fed with CDAHFD (and treated with adenylated miR-12135 (adenylated) (n = 5). At days 5, 8, 12, 15, and 19, the mice were given each miR (3 nmol/mouse intraperitoneally in Atelogene [Koken, Tokyo, Japan]). At day 20, all mice were sacrificed under isoflurane vapor. The organs were harvested and used for analysis. No mouse was excluded in any group. All animals were randomly assigned (chosen alternately) to each group and not blinded. The experimental unit for the *in vivo* studies is the mouse. ALT/AST and TG levels were assessed by using Transaminase test Wako (Fujifilm) and TG test Wako (Fujifilm) in accordance with its manufacture’s protocols.

For the NASH model study (long-term miR-12135 treatment), 5-week-old male C57BL/6J mice (3 groups of 7 mice each; 21 in total) were purchased from Kyudo (Saga, Japan) for the study and acclimated for 1 week to housing conditions and an MF diet (KBT Oriental). At 6 weeks of age, the mice were randomly divided into 3 groups: Gp1 was fed an MF diet (n = 7), Gp2 was fed a CDAHFD and treated with non-targeting RNA (n = 7), and Gp3 was fed a CDAHFD and treated with miR-12135 (n = 7). Treated mice were intraperitoneally administered RNA (3 nmol/mouse) dissolved in Atelogene (Koken) (Figure 7A). On the final day, all mice were sacrificed under isoflurane vapor anesthesia. The organs were harvested and used for analyses. No mice were excluded from each group. All mice were randomly assigned (chosen alternately) to each group, and we were not blinded to this assignment. The experimental unit for our *in vivo* studies was a mouse. In the NASH model study (long-term siITGA11RNA treatment), 5-week-old male C57BL/6J mice (3 groups of 7 mice each; 21 in total) were purchased from Kyudo for the study and acclimated for 1 week to housing conditions and an MF diet (KBT Oriental). At 6 weeks of age, the mice were randomly divided into 3 groups: Gp1 was fed an MF diet (n = 7), Gp2 was fed a CDAHFD and treated with non-targeting RNA as Scr-siRNA (n = 7), and Gp3 was fed a CDAHFD and treated with siITGA11RNA (siRNA ID: 154044; Silencer™, Thermo Fisher Scientific) (n = 7). Treated mice were intraperitoneally administered RNA (3 nmol/mouse) dissolved in Atelogene (Koken) (Figure 8A). On the final day, all mice were sacrificed under isoflurane vapor anesthesia. The organs were harvested and used for analyses. No mice were excluded from each group. All mice were randomly assigned (chosen alternately) to each group, and we were not blinded to this assignment. The experimental unit for our *in vivo* studies was a mouse.

#### Liver fibrosis model (BDL)

In the bile duct ligation (BDL) model study (miR-12135 treatment), 7-week-old male C57BL/6J mice (3 groups of 7 mice each; a total of 14 BDL-treated and 7 sham-treated mice) were purchased from Kyudo (Saga, Japan) for this study. In the BDL operation, the abdomens of mice were shaved with an electric clipper, and their epidermis was disinfected with povidone-iodine prior to making an incision with scissors in the skin and muscle layer, approximately 1 cm below the xiphoid process. The duodenum was then grasped lightly with ring tweezers, pulled out, and ligated into the upper region of the bile duct using two sutures.

Gp1 mice were sham-operated (n = 7), Gp2 mice were subjected to BDL and treated with non-targeting RNA (n = 7), and Gp3 mice were subjected to BDL and treated with miR-12135 (n = 7). Treated mice were intraperitoneally administered RNA (3 nmol/mouse) dissolved in Atelogene (Koken) (Figure 9A). BDL-treated animals were randomly assigned (chosen alternately) to each group, with equivalent average body weights maintained. We were not blinded to this assignment, and all the mice were fed an MF diet throughout the study. On the final day, all mice were sacrificed under isoflurane vapor anesthesia. The organs were harvested and used for analyses. No mice were excluded from any group, but 2 mice (both in the control group) died before sacrifice.

#### Fluorescence microscopy analysis

Harvested liver tissue was fixed by 4% PFA (Fujifilm, Tokyo, Japan) for 1 week and paraffin-embedded by Kyodo Byori (Kobe, Japan). Deparaffinization of the sections was done with Lemozol™ (Fujifilm, Tokyo, Japan) (for 10 min, 3 times) and EtOH (for 5 min, 3 times). Antigen retrieval was carried out using Immunosaver™ (Nissin EM, Tokyo, Japan) diluted by deionized water in accordance with manufacturer’s protocol and heated (95°C, 45 min). After the blocking step by 1% FBS 0.1% sodium azide containing PBS for 1 h, the section was incubated with primary antibodies (1:200 in 1% FBS 0.1% sodium azide containing PBS, overnight). After washing thrice with PBS, the section was incubated with the secondary antibody (1:1000 in 1% FBS 0.1% sodium azide containing PBS supplemented with Hoechst33342 [10000 dilution]; Thermo Fisher Scientific) for 1 h. After washing thrice with PBS, slides were treated with Vecta shield™ (Vector Laboratories, Burlingame, CA, USA) and observed using the BZ-X700 (Keyence) under 40× magnification. Patient samples were purchased from US BIOMAX (Cat#BC03117a). Fluorescence intensity was evaluated by Image J (NIH, Bethesda, MD, USA). Photo images had their brightness and contrast adjusted using PowerPoint (Microsoft, Seattle, WA).

#### *In vitro* study for IF study

In the fluorescence microscopy analysis for the effect of equol, LX2 cells were seeded in a glass bottom dish (Mastunami, Osaka, Japan) and precultured for 24 h in 2% FBS DMEM. Afterwards, LX2 cells were treated with equol (10 μM) for 24 h and treated with TGF-β (5 ng/mL) for 48 h and fixed by 2% paraformaldehyde (PFA) (Fujifilm, Tokyo, Japan). The cells were also treated with 1% FBS-Phosphate Buffered Saline (PBS)-0.2% Tween and incubated with primary antibody (1:200 in 1% FBS 0.1% sodium azide containing PBS for 0.75 h) and washed with PBS 3 times. Dishes were treated with secondary antibody (1;300 in 1% FBS 0.1% sodium azide containing PBS supplemented with Hoechst33342 [10000 dilution Thermo Fisher Scientific] for 0.75 h.). After washing thrice with PBS, the cells were observed using the BZ-X700 (Keyence) under 40× magnification.

#### ATPlite assay

In the viable cell analysis of LX2 cells for the effect of miR-12135, LX2 cells were seeded in a 96-well plate and precultured for 24 h in 2% FBS DMEM. After that, LX2 cells were treated with miR-12135 mimic using the RNAi max for 24 h and 48 h and the viable cell numbers were assessed by ATPlite one step (Perkin Elmer) as followed by manufacture’s protocol.

#### *In vitro* study for miRNA and knockdown

Human cell line derived from cervical cancer, HeLa cells (ATCC, Manassas, VA, USA), were cultured in DMEM supplemented with penicillin-streptomycin and 10% FBS under 100% humidity and 5% CO_2_ at 37°C. Hepatic stellate cells (LX2 cells) (Merck, German, Darmstadt) were maintained in DMEM supplemented with penicillin-streptomycin and 2% FBS under 100% humidity and 5% CO_2_ at 37°C.

In the NGS analysis for adenylated micro RNAs, HeLa cells were seeded in a 10-mL dish and precultured in 10% FBS DMEM for 24 h, which was then replaced by 2%FBS DMEM with or without 10 μM equol supplementation. The cells were cultured for 24 h and harvested using TRI Reagent (Cosmo Bio, Tokyo, Japan). Hiseq was performed using the Cell innovator (Fukuoka, Japan).

In the real-time PCR analysis of HeLa cells, HeLa cells were seeded in a 12-well plate and precultured for 24 h in 10% FBS DMEM. After preculture, cells were treated with 10 nM miR-12135 mimic and adenylated miR-12135 mimic using the RNAi max (Thermo Fisher Scientific, Added 200 μL of Opti-MEM 1.7 mL + RNAi max 20.4 μL + miR-12135 mimic [10 μM] 10.2 μL) for 48 h and harvested using the TRI Reagent.

In the real-time PCR analysis of LX2 cells for the effect of equol, LX2 cells were seeded in a 12-well plate and precultured for 24 h in 2% FBS DMEM. Afterwards, LX2 cells were treated with equol (10 μM) for 24 h and treated with TGF-β (5 ng/mL) for 48 h and harvested using the TRI Reagent.

In the real-time PCR analysis of LX2 cells for the effect of SiRNA-ITGA11, LX2 cells were seeded in a 12-well plate and precultured for 24 h in 2% FBS DMEM. Afterwards, LX2 cells were treated with SiRNA-ITGA11 using the RNAi max (Added 200 μL of Opti-MEM 1.7 mL + RNAi max 20.4 μL + SiRNA-ITGA11 [5 μM] 10.2 μL) for 24 h and treated with TGF-β (5 ng/mL) for 48 h and harvested using the TRI Reagent.

In the WB analysis of LX2 cells for the effect of miR-12135 alone, LX2 cells were seeded in a 12-well plate and precultured for 72 h in 2% FBS DMEM. Afterwards, LX2 cells were treated with miR-12135 mimic using the RNAi max for 48 h and harvested using the sample buffer.

In the real-time PCR analysis of LX2 cells for the effect of miR-12135 and TGF-β, LX2 cells were seeded in a 12-well plate and precultured for 24 h in 2% FBS DMEM. Afterwards, LX2 cells were treated with miR-12135 mimic using the RNAi max for 24 h and treated with TGF-β (5 ng/mL) for 48 h and harvested using the TRI Reagent.

#### miRNA inhibitor experiments

In the miR-12135 inhibitor assay, LX2 cells were seeded at glass bottom dish (2%FBS DMEM, 1 mL/dish) and precultured for 24 h. After preculture, cells were treated with NC mimic or miR-12135 inhibitor (10 nM, Ambion, mirVana™, Cat#4464084) by using RNAimax ([RNAimax 20.4 μL + Optimem 850 μL] + [miR-12135 inhibitor (10 μM) 10.2 μL + Optimem 850 μL] 200 μL/well). Afterwards, 6 h later, cells were treated with 10 μM equol, and 24 h later, cells were treated with TGF-β (5 ng/mL) for 48 h. After treatment, cells were fixed using 2% PFA and treated with 1% FBS-PBS-0.2% Tween and incubated with primary antibody (1:200 in 1% FBS 0.1% sodium azide containing PBS for 0.75 h) and washed by PBS 3 times. Dishes were treated with secondary antibody (1:300 in 1% FBS 0.1% sodium azide containing PBS supplemented with Hoechst33342 [10000 dilution Thermo Fisher Scientific] for 0.75 h). After washing thrice with PBS, the cells were observed by using BZ-X700 (Keyence) under 40× magnification.

#### In the miR target luciferases assay

HeLa cells were seeded in a 96-well plate and precultured for 24 h. The medium was removed and replaced with 2%FBS DMEM. Afterwards, we added luciferase vector with the 3′UTR of ITGA11 with or without the target predicted sequence of miR-12135 (NM_001004439.1, pEZX-MT06 HmiT130500-MT06, from GeneCopoeia) and control luciferase vector without the 3’UTR of ITGA11 (HmiT130500-MT06, from Gene Copoeia) by using Fugene 6 and transfected with miR-12135 mimic (10 nM) or Negative control (NC) mimic (10 nM) by RNAimax. After 48 h, the supernatants were removed, ONE-Glo™ EX Luciferase Assay Substrate was added, and the cells were measured using the plate reader Envision™.

### Quantification and statistical analysis

All data are shown as mean ± standard error of the mean (SEM). Significant differences between experimental variables were evaluated via Student's t-test (for 2 groups; unpaired two-sided). Multiple comparisons (more than 2 group) were performed by the one-way analysis of variance followed by Dunnett’s test. All statistical analyses were performed by GraphPad 8.0 (GraphPad Software). *P* < 0.05 was considered to indicate a significant difference.
