# Supplementary material for: miR-12135 ameliorates liver fibrosis accompanied with the downregulation of integrin subunit alpha 11
Source: iScience. 2023 Dec 14;27(1):108730. doi: 10.1016/j.isci.2023.108730 (PMC10792239; doi:10.1016/j.isci.2023.108730)
Supplement: Document S1. Figures S1–S7 and Tables S1–S3 [file mmc1.pdf]

iScience, Volume *27*

Supplemental information

miR-12135 ameliorates liver fibrosis accompanied with the downregulation of integrin subunit alpha 11

Motofumi Kumazoe, Emi Miyamoto, Chihiro Oka, Miyuki Kondo, Ren Yoshitomi, Hiroaki Onda, Yu Shimada, Yoshinori Fujimura, and Hirofumi Tachibana

A

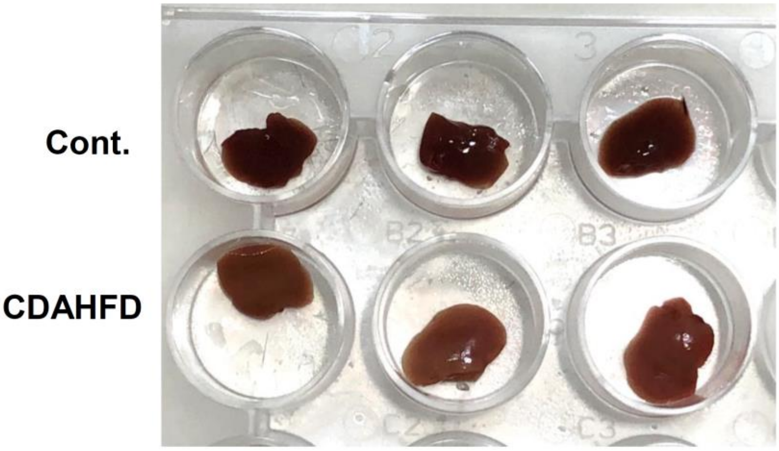

B

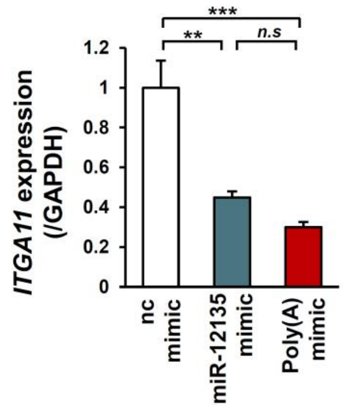

SI Fig1 miR-12135 suppressed ITGA11 expression, related to Figure 3.

(**A**) Liver tissue images of the mice fed with the MF diet and choline-deficient, L-amino acid-defined, high-fat diet (CDAHFD). (**B**) HeLa cells were transfected with the indicated miR for 48 h, and were evaluated via qRT-PCR (*n* = 4). All data are shown as the mean ± SEM. $^{**}P < 0.01$. $^{***}P < 0.001$.

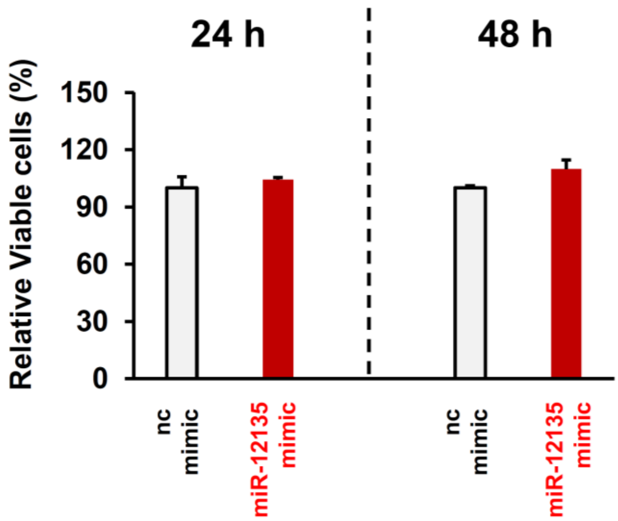

SI Fig2 The effect of miR-12135 on viable cell numbers of LX2 cells, related to Figure 4.

LX2 cells were transfected with the indicated miR (20 nM) for 24 or 48 h, and viable cell numbers after 48 and 96 h were determined by ATPlite assay (n = 4). All data are shown as the mean ± SEM.

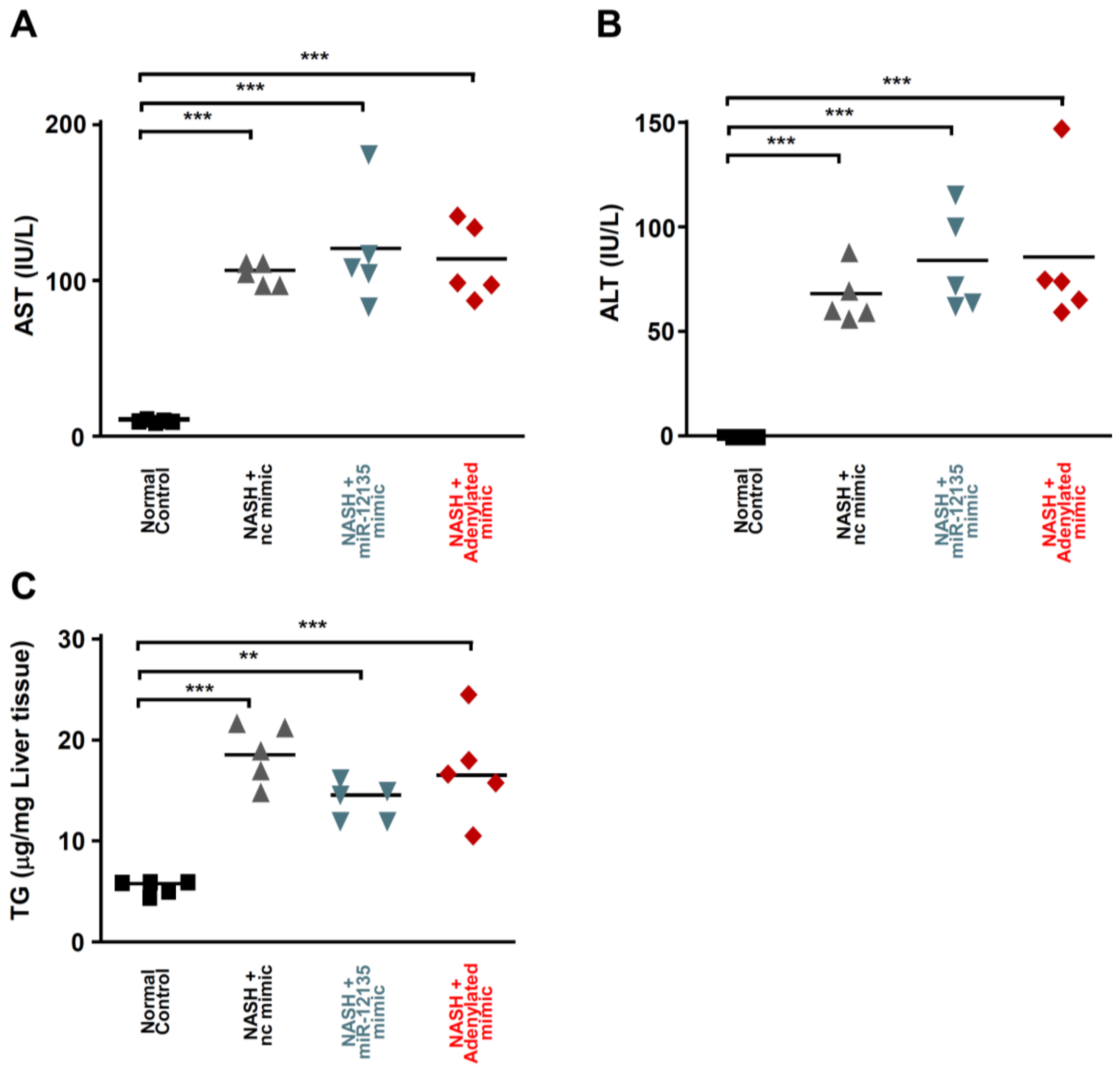

SI Fig3 miR-12135 did not affect TG, ALT and AST levels in CDAHFD model mice, related to Figure 5.

A scheme of the choline-deficient, L-amino acid-defined, high-fat diet (CDAHFD) mouse model was shown in Fig5A. To assess the in vivo activity of miR-12135 and adenylated miR-12135 in liver fibrosis, male C57BL/6J mice (5 per group) were fed CDAHFD and mice were then given intraperitoneal injection of miR-12135 and adenylated miR-12135 or negative control miRNA (3 nmol/mouse) dissolved in atelogene. Mice are fed for 20 days with CDAHFD. Serum AST/ALT levels and liver TG levels were determined. ***$P < 0.001$.**$P < 0.01$.

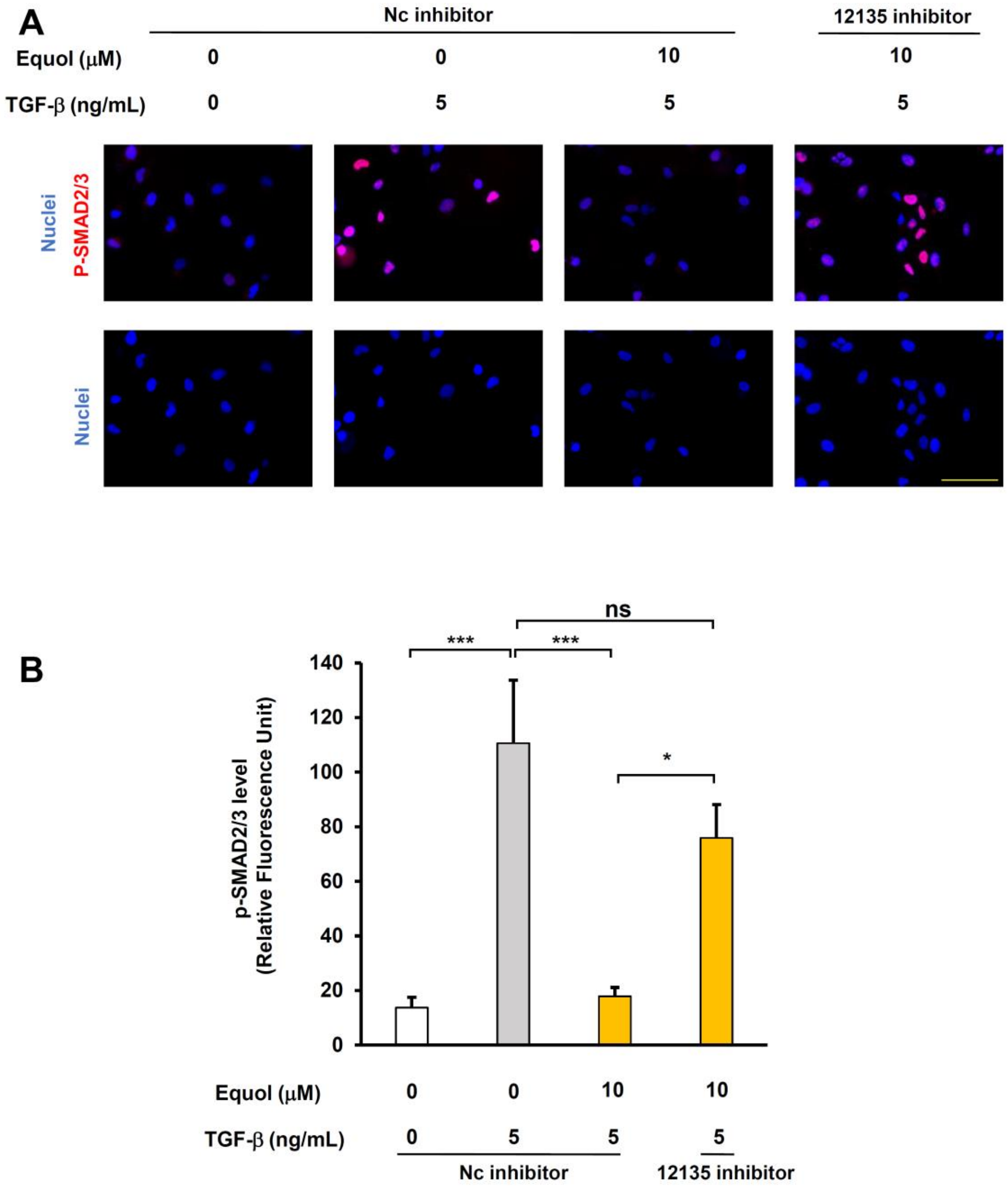

SI Fig4 Equol suppressed TGFβ-induced p-SMAD2/3 increase though miR12135-depdent mechanisms, related to Figure 6.

LX2 cells were treated with miR-12135 (10 nM) inhibitor for 6 h, pretreated with equol (10 μM), and treated with TGF-β (5 ng/mL) for 48 h (n = 14, 14, 13, 24). Nuclei P-SMAD2/3 levels were determined by the fluorescence microscopy with using anti-SMAD2/3 antibody. ***$P < 0.001$, *$P < 0.05$. (Bar 100 μm).

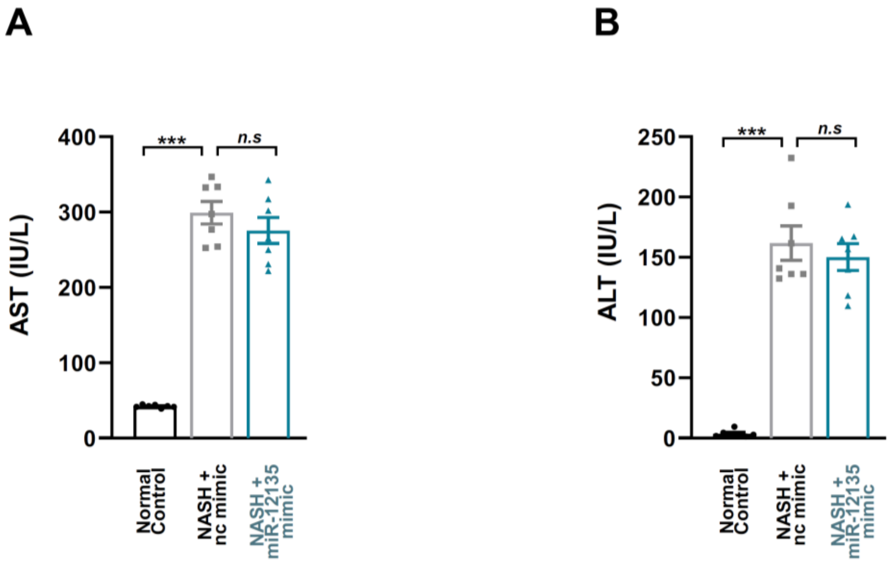

SI Fig5 miR-12135 did not affect ALT and AST levels in CDAHFD model mice, related to Figure 7.

A scheme of the choline-deficient, L-amino acid-defined, high-fat diet (CDAHFD) mouse model was shown in Fig7A. To assess the in vivo activity of miR-12135 in liver fibrosis, male C57BL/6J mice (7 per group) were fed CDAHFD and mice were then given intraperitoneal injection of miR-12135 or negative control miRNA (3 nmol/mouse). Serum AST/ALT levels were determined. ***$P < 0.001$.

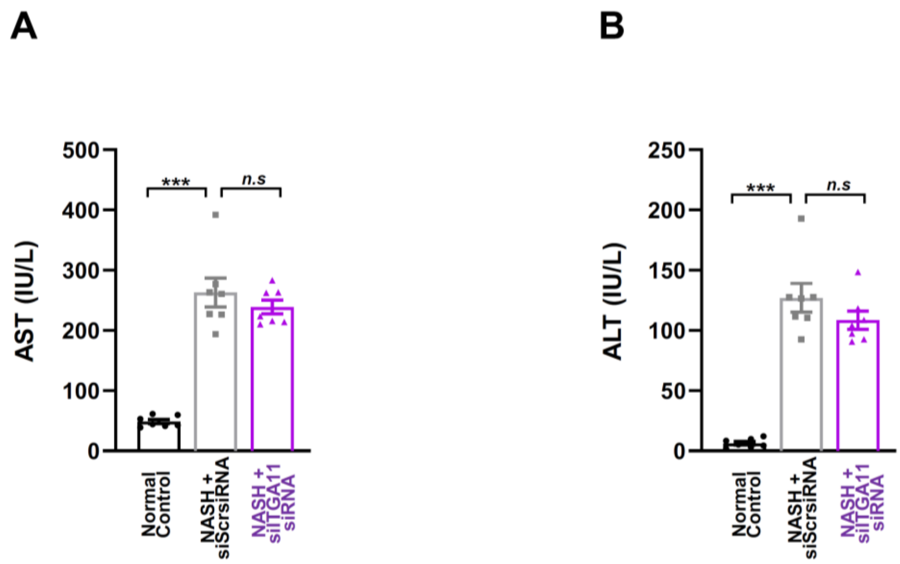

SI Fig6 SiITGA11RNA treatment did not affect ALT and AST levels in CDAHFD model mice, related to Figure 8.

A scheme of the choline-deficient, L-amino acid-defined, high-fat diet (CDAHFD) mouse model was shown in Fig8A. To assess the in vivo activity of SiITGA11RNA treatment in liver fibrosis, male C57BL/6J mice (7 per group) were fed CDAHFD and mice were then given intraperitoneal injection of SiITGA11RNA treatment or negative control RNA (3 nmol/mouse). Serum AST/ALT levels were determined. ***$P < 0.001$.

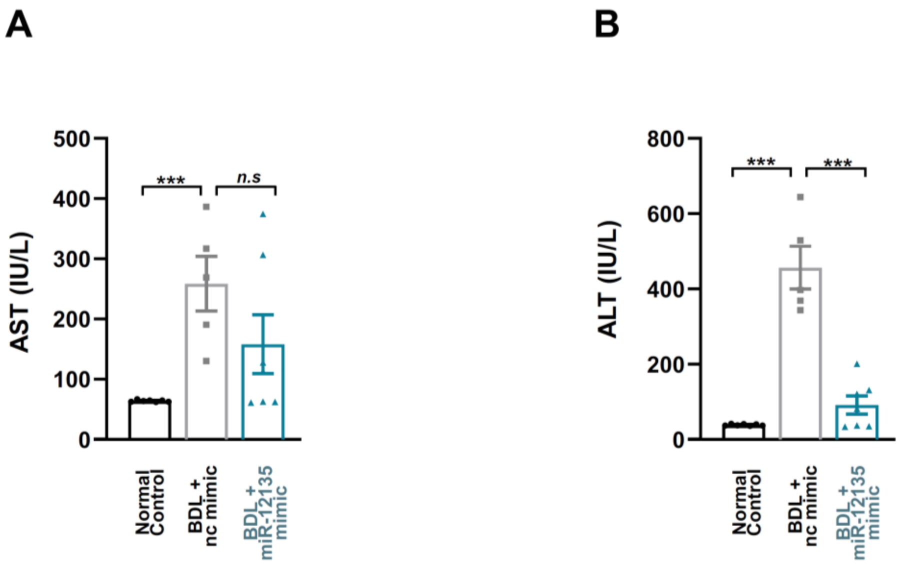

SI Fig7 The effect of miR-12135 of the ALT and AST levels in BDL model mice, related to Figure 9.

A scheme of BDL mouse model was shown in Fig9A. To assess the in vivo activity of miR-12135 in liver fibrosis, male C57BL/6J mice (7 per group) subjected to the BDL operation and mice were then given intraperitoneal injection of miR-12135 or negative control miRNA (3 nmol/mouse). Serum AST/ALT levels were determined. ***$P < 0.001$.

SI Table 1

The expression levels of ASMA and ITGA11 were determined by IHC analysis, related to Figure 3.

No	Age	Sex	Status	ASMA	ITGA11
No1	48	F	Cirrhosis	43.61	21.12
No2	57	F	Cirrhosis	125.71	72.79
No3	52	M	Cirrhosis	27.09	16.03
No4	49	M	Cirrhosis	22.43	17.42
No5	48	M	Cirrhosis	28.8	18.89
No6	67	M	Cirrhosis	115.78	60.41
No7	53	M	Cirrhosis	28.92	20.74
No8	55	M	Cirrhosis	45.96	33.25
No9	35	M	Cirrhosis	16.15	12.88
No10	34	M	Cirrhosis	21.04	17.57
No11	63	M	Cirrhosis	26.28	14.5
No12	57	F	Cirrhosis	56.18	25.37
No13	37	M	Cirrhosis	24.52	12.61
No14	40	M	Cirrhosis with bile duct proliferation	39.57	20.38
No15	54	M	Cirrhosis with bile duct proliferation	39.94	24.8
No16	55	M	Cirrhosis	40.9	27.79

SI Table 2 miRNA sequence, related to Figure 3.

**miR-12135**	5' UAAAGGUUUGUUUGUAAAtt 3'
	5' UUUACAAACAAACCUUUAtt 3'
**polyadenylated miR-12135**	5' UAAAGGUUUGUUUGUAAA AAAAAAAAAtt 3'
	5' UUUACAAACAAACCUUUAtt 3'

SI Table 3 Primer sequence, related to Figure 1 and Figure 3.

Gene	Forward	Reverse
**ITGA11**	**CAGCTCGCTGGAGAGATACG**	**TTACAGGACGTGTTCGCCTC**
**COL1A1**	**AGGGCCAAGACGAAGACATC**	**GTCGGTGGGTGACTCTGAGC**
**ACTB**	**TGGCACCCAGCACAATGAA**	**CTAAGTCATAGTCCGCCTAGAAGCA**
**FN1**	**GGAGAATTCAAGTGTGACCCTCA**	**TGCCACTGTTCTCCTACGTGG**
